# Using machine learning to predict processes and morphometric features of watershed

**DOI:** 10.1038/s41598-023-35634-2

**Published:** 2023-05-25

**Authors:** Marzieh Mokarram, Hamid Reza Pourghasemi, John P. Tiefenbacher

**Affiliations:** 1 Department of Geography, Faculty of Economics, Management and Social Sciences, Shiraz University, Shiraz, Iran; 2 Department of Soil Science, College of Agriculture, Shiraz University, Shiraz, Iran; 3 Department of Geography, Texas State University, San Marcos, TX USA

**Keywords:** Environmental sciences, Natural hazards, Engineering

## Abstract

The research aims to classify alluvial fans’ morphometric properties using the SOM algorithm. It also determines the relationship between morphometric characteristics and erosion rate and lithology using the GMDH algorithm. For this purpose, alluvial fans of 4 watersheds in Iran are extracted semi-automatically using GIS and digital elevation model (DEM) analysis. The relationships between 25 morphometric features of these watersheds, the amount of erosion, and formation material are investigated using the self-organizing map (SOM) method. Principal component analysis (PCA), Greedy, Best first, Genetic search, Random search as feature selection algorithms are used to select the most important parameters affecting erosion and formation material. The group method of data handling (GMDH) algorithm is employed to predict erosion and formation material based on morphometries. The results indicated that the semi-automatic method in GIS could detect alluvial fans. The SOM algorithm determined that the morphometric factors affecting the formation material were fan length, minimum height of fan, and minimum fan slope. The main factors affecting erosion were fan area (*A*_*f*_) and minimum fan height (*H*_*min-f*_). The feature selection algorithm identified (*H*_*min-f*_), maximum fan height (*H*_*max-f*_), minimum fan slope, and fan length (*L*_*f*_) to be the morphometries most important for determining formation material, and basin area, fan area, (*H*_*max-f*_) and compactness coefficient (*C*_*irb*_) were the most important characteristics for determining erosion rates. The GMDH algorithm predicted the fan formation materials and rates of erosion with high accuracy (R^2^ = 0.94, R^2^ = 0.87).

## Introduction

Most of the studies on alluvial fans have focused on the physical and mechanical properties of alluvial deposits for concrete production^1^. Few have focused on the distribution and morphometry of alluvial fans relative to geomorphology^2–4^. Alluvial fan sedimentary dynamics are influenced by various factors: the geology of upstream lands that generate sediments for alluvial fans, landslides, and glaciers^5^. The role of morphometry features in sedimentation processes is investigated in^6^. Investigation of morphometric characteristics of alluvial fans enables prediction of superficial activities like erosion and deposition as well as internal activities like tectonics^7^. Other studies conducted in the field of investigating the relationship between form and process can be referred to as^8–10^.

Topographic and digital elevation model (DEM) data can be used to study alluvial fans. By measuring morphometric factors like elevation, slope, curvature, aspect, texture, morphology, and drainage patterns in a DEM, various geomorphological features such as different landform types (like alluvial fans) can be analyzed (Fig. S1)^11^.

Among the various forms of alluvium, alluvial fans are prominent geomorphological features that are formed in any type of climate, especially in arid and semi-arid climates^12^. Alluvial fans are located in the downstream parts of the watershed, where the slope decreases^13–16^.

At least 5 main factors affect alluvial fan processes^12,17^. They include catchment lithology, basin shape, conditions adjacent to the alluvial fan, climate, and tectonic activity. Field investigation of alluvial fans in forests or in desert conditions can be difficult and tedious. It is also challenging and time-consuming to study alluvial fans over large regions^18^.

Using GIS, remote sensing, and DEMs enables faster and easier identification and study of alluvial fans. Remote sensing was used by^18^ to study the morphology of deformed alluvial fans resulting from fault processes in Bole Basin, northern Tian Shan. According to^19^, there is a significant relationship between the shape, the direction, and the amount of erosion in Hellas Basin, Mars. The linear regression method was used by^20^ to examine the relationship between alluvial cone soil quality and alluvial cone morphometry. Among their studies, they found a significant correlation between soil quality and morphometric characteristics.

Predictions of the composition and forms of alluvial fans have been improved by the rapidly developing science of artificial intelligence and neural networks to extract environmental data^21–23^. These methods more clearly reveal the morphometric features affecting watershed processes. The artificial neural network (ANN) algorithm can be used to predict processes using watershed-based morphometric parameters^24,25^ used ANN and logistic regression (LR) to study alluvial fans in Calabria, Italy. LR and ANN have shown that lithology affects the dynamics of alluvial fans^26^. investigated the effects of climate, geology, and topography on the alluvial fan morphometry and found that increased precipitation changes morphometry and lithology affect morphometry^13^, using the self-organizing map (SOM) method to study the morphometry of a watershed, found a strong relationship between drainage status and the morphometry of alluvial fans.

ANN is a predictive model used in many geomorphological studies^27^, but it has not yet been used to study alluvial fan morphometry. This study aims to determine alluvial morphometry and use the SOM algorithm and to identify the morphometric parameters that best explain alluvial fans and erosion rates. Input data is selected using the feature selection algorithm, and formation material and erosion rates are predicted using the Group Method of Data Handling (GMDH) algorithm. This study is one of the few studies that combine three neural network models—SOM, GMDH, and feature selection—to investigate and predict alluvial fan morphometry and its relationship to the upstream watershed morphology. The use of neural networks to investigate the morphometric properties of alluvial fans has not been studied. Among neural networks, the GMDH neural network has an advantage over others due to the use of polynomials to study processes^28,29^.

Group Classification of Numerical Data (GMDH) is a neural network model used for identifying, predicting, and classifying data. The GMDH technique combines self-organizing systems, control theory, and information science^30^. Due to the regularity of the processes, the GMDH method overcomes the statistical weaknesses of neural networks. To preserve the topological properties of the input space, SOM neural networks use a neighborhood function^31^. Many neural network methods use a small number of neurons to implement the network. Unlike SOM networks, SOM networks preserve the topological features of the data^32^. In the SOM method, unlike classification methods that analyze data based on classes, data is analyzed without considering class labels. This leads to more accurate data clustering^33^.

This approach can help identify and even predict the processes in the watershed based on the morphometric characteristics of alluvial fans. The research steps are generally as follows:

1. Extraction of 25 morphometric characteristics of alluvial cones in GIS
2. Classification of alluvial fans based on morphometric characteristics using SOM
3. Selecting the most important morphometric parameters effective in determining the rate of erosion and lithological features using principal component analysis (PCA), Greedy, Best first, Genetic search, Random search as feature selection algorithms
4. Using the GMDH algorithm to predict erosion and lithology features based on morphometric features

Organizing this paper is as follows:the characteristics of the study area in this study are presented in “Materials and methods” section. In addition, this section describes the methods used to predict soil erosion and lithology features. The results of the research are presented in “Results and discussion” section. Finally, “Conclusion” section provides conclusions.

## Materials and methods

In “Case study” section describes the case study of the research. In “Semi-automatic method to extract alluvial fan and data characteristics” section describes a semi-automated method for extracting alluvial fan characteristics and data characteristics. The methods that are used are explained in “Modeling methods” section, including the "SOM" method, "Feature selection algorithm", "GMDH" algorithm, and "Validation model".

### Case study

**Figure 1:**
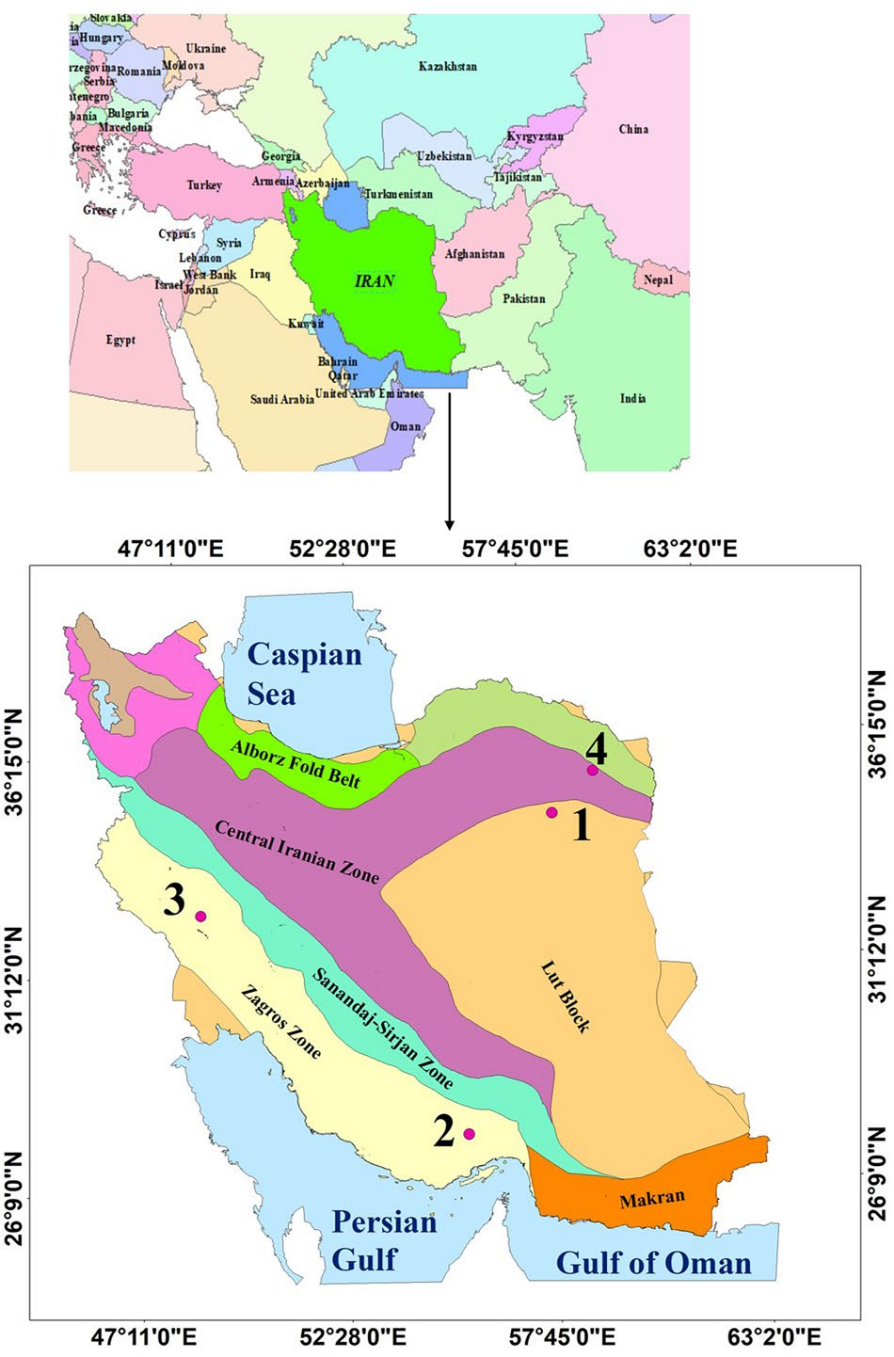
The locations of the study areas: (1) Central Desert watershed, (2) Mehran watershed, (3) Central Desert watershed, and (4) Karun watershed.

**Figure 2:**
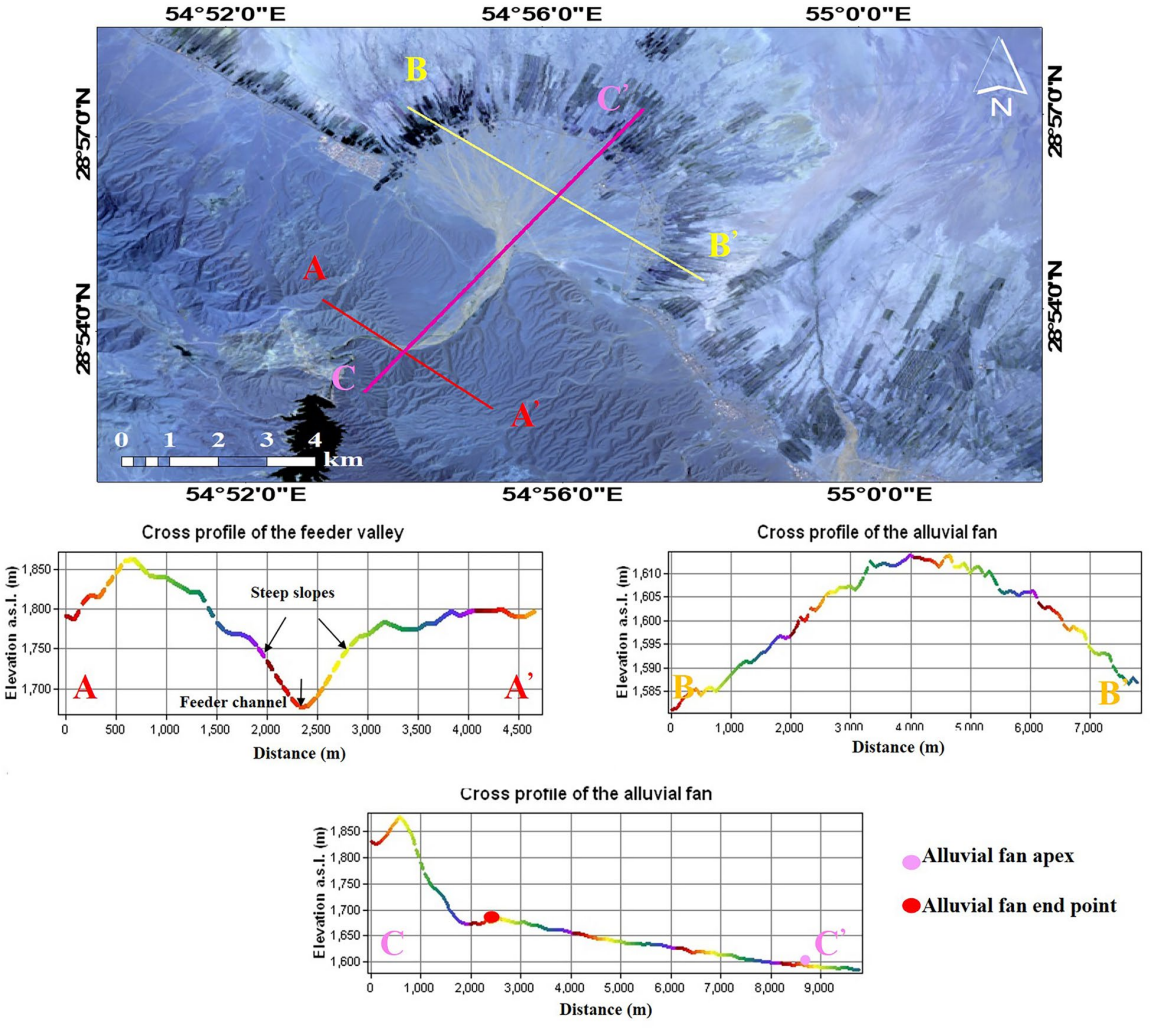
Morphological status of a large alluvial fan in the Mehran watershed (**a**) the alluvial fan from satellite images (**b**) topographic characteristics of the profiles AA’, BB’, CC’.

Four watersheds—two Central Desert watersheds, the Mehran watershed, and the Karun watershed—located in northwestern, northeastern, and southern Iran (Fig. 1), were selected for a study of the effects of formations and climates on morphometrics using the morphometric characteristics of alluvial fans (Fig. 2). Sample 1 is located in the Lut Block of geomorphological regions. Samples 2 and 3 are in the Zagros zone. And sample 4 is located in the Central Iran region.

The weather in this region is scalding, and humid in summers and sometimes the temperature exceeds 52°. The average annual temperature in this region is about 27°. In this basin, about 9 months of the year, there is no significant rainfall and most of the rainfall occurs once or twice. In the same rare cases, the rainfall is often like torrential spring rains and causes a lot of damage. The relative humidity in the Persian Gulf is mostly high and fluctuates between 20 and 100%. The total annual rainfall in most parts of the region is less than 300 mm.

In Karun watershed, rainfall varies are from about 150 mm in the southern regions to more than 1000 mm per year in the northern highlands and eastern regions, and its precipitation regime in the Mediterranean. In Central Kavir watershed, the average total rainfall of stations in the basin is 256.5 mm. The average annual temperature of the whole period in the central basin is 13.99°, the absolute maximum of the warmest months of the period is 48°, the absolute minimum of the coldest months of the period is − 35°.

The Zagros zone includes the highlands of western and southwestern Iran (Zagros) and consists of Lorestan, Khuzestan and Fars regions. The existence of huge gas and oil fields has made the Zagros one of the most oil-rich sedimentary basins in the world. The climate in this mountainous massif is strongly influenced by the altitude factor, so that with increasing altitude decrease in temperature, and changes precipitation conditions. Rainfall in this zone is between 400 and 1000 mm. As shown in Fig. 1 alluvial fans in different zones are morphometrically different from each other, which indicates the impact of the formation material and different climatic conditions.

### Semi-automatic method to extract alluvial fan and data characteristics

The morphometry characteristics of alluvial fans, have been analyzed in other studies in which alluvial fans are identified as landforms created by accumulating sediments transported from mountains. The sedimented landforms are semi-conical, with slopes decreasing with distance from the mountain^34^. The morphometrics of an alluvial fan can be a semi-conical surface. In the GIS algorithm, a conical surface is made by joining a series of profiles radiating from the fan apex. The channels are mapped, the radial slopes are mapped, and the semi-conical surface is interpolated (Fig. 2).

The alluvial fan boundary is located at the intersection of the alluvial fan radius with the fan edge (radial profile in Fig. 2). The shapes of alluvial fans vary depending on the bedrock, the shape of the watershed, climate, and tectonic activities, alluvial fans have different shapes. The average slope of alluvial fans is between 2° and 35°^35^. The slope of the distal portions of alluvial fans varies between 5° in dry areas and 1° in wet areas^36^. The spatial extents of alluvial fans are affected by watershed size: large alluvial fans are formed at the bottom of large watersheds^37^.

**Figure 3:**
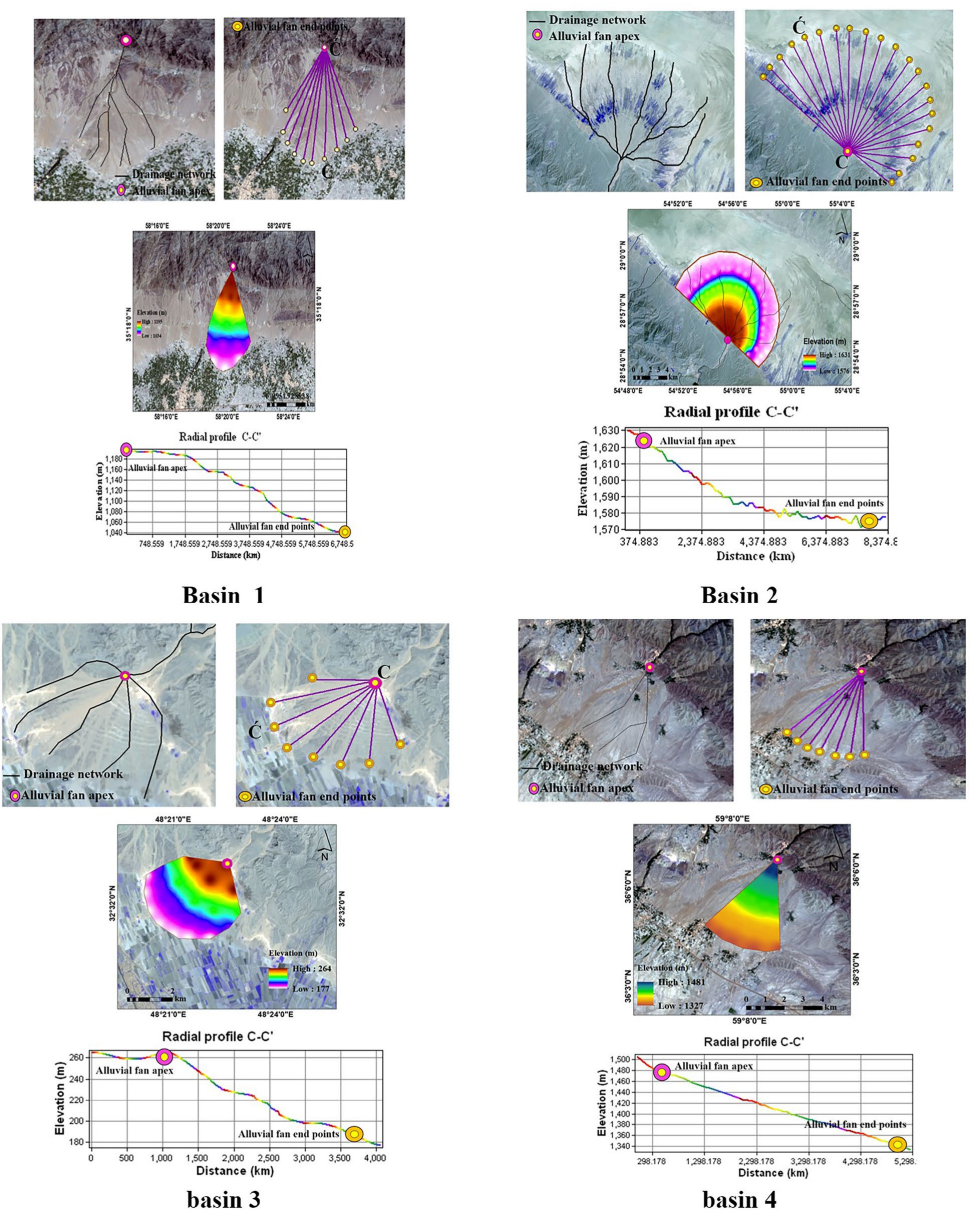
The steps extract the alluvial fan in the GIS algorithm.

**Figure 4:**
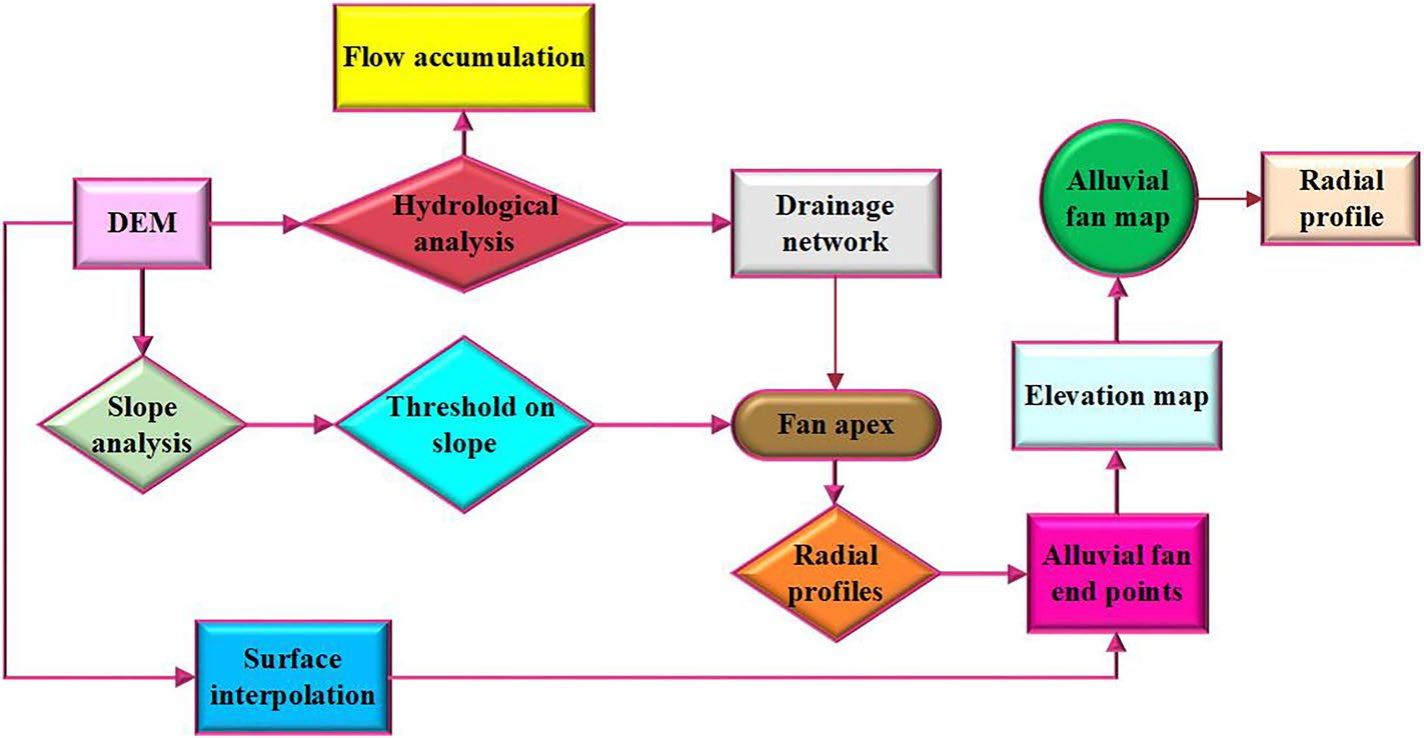
Steps to extract the alluvial fans from DEMs.

Radial profile analysis is mainly based on a fixed or variable minimum slope threshold that examines slope changes along each fan (slope threshold is defined by trial and error or training on a representative alluvial fan). The semi-conical surface of the alluvial fan is used to cut the radial profile. The apex is the location of the input of sediment to the alluvial fan (Fig. 3). The apex is the entrance of the stream onto the alluvial fan. Topographic surfaces were created for the four alluvial fans from the radial profiles and data extracted from the DEMs (Fig. 4).

Twenty-four morphometric features were calculated for each alluvial fan (Table S1).

### Modeling methods

The SOM algorithm was used to classify the morphometric parameters and the feature selection algorithm was used to select the parameters that are most important for predicting formation materials and alluvial erosion rates. The GMDH algorithm was used to predict these two outcomes. A summary of the research steps is shown in Fig. S2.

#### Self-organizing map (SOM)

The SOM neural networks are implemented in two stages: training and mapping. During the training phase, input samples are used to create the mapping. In the mapping phase, the original input vectors are automatically classified^38^. The self-organizing map consists of neurons. Each neuron is characterized by a weight vector with dimensions equal to the input data dimensions and its position in the mapping space. Neurons are usually arranged in a regular space in the form of a hexagonal or rectangular network. Self-organizing mapping describes a high-dimensional input space into a low-dimensional mapping. To place a vector from the input data space on the map, SOM finds the neuron with the closest weight vector to the input data space^39^. After the nearest neuron is determined, the neuron's weight vector value is updated according to the input data. In this method, it is very common to use the U matrix. The value of a neuron in the U matrix is the average distance between it and its nearest neighbors. Unlike most old methods that use a simple algorithm to solve problems, this method takes advantage of learning from examples. Saman's neural network algorithm is suitable for data clustering^40^. It converts non-linear statistical relationships between input data into simple geometric relationships, which is an invariant recursive regression relationship, as the regression is performed recursively by presenting each sample. The Self-organizing mappings differ from other artificial neural networks in that they use a neighborhood function to preserve the input space characteristics^41^.

SOM composed of neural cells that attach to adjacent cells through neighborhood relations^42–44^. In the training step, the *x* vector is selected from the input set and all weight vectors are calculated using distance measurements like Euclidean distance^45^. A neuron whose weight vector is close to the input variable *x* is called the “best unit” (BU) (Eq. 1).

1 $$||x - m_{c} || = \min \{ ||x - m_{i} ||\}$$

where *x* is the input vector and *m* is the weight vector.

After finding the BU, the weight vectors are updated and moved closer to the input vector using the SOM update rule for the unit weight vector (Eq. 2):

2 $$m_{i} (t + 1) = m_{i} (t) + \alpha (t)h_{ci} (t)[x(t) - m_{t} (t)]$$

In Eq. (2), *α(t)* depicts the learning rate, while, *x(t)* is to show the input data at time t. Moreover, *m(t)* shows the output location and *c*_*i*_*(t)* depicts the neighborhood kernel.

#### Feature selection algorithm

After data pre-processing, the Principal component analysis (PCA), Greedy, Best first, Genetic search, Random search as feature selection algorithms were used to select the important parameters in WEKA software^46^. The cross-validation process with ten folds was used to define the training and test sets. Filtering was achieved with the correlation-based feature selection (CFS) algorithm^47^. CFS is a simple filter algorithm that ranks feature subsets according to a correlation-based heuristic evaluation function.

#### Group method of data handling (GMDH) method

Based on the quadratic transfer function, the GMDH network builds a function in a network. Multivariate processes are often modeled and predicted using GMDH. GMDH neural networks contain neurons arising from quadratic polynomials. In GMDH neural networks, the most common neurons in each layer can only communicate with neurons in the previous layer. In order to generalize GMDH neural networks, it is necessary to remove the requirement that the adjacent layer be used to construct the next layer^48^.

The GMDH neural network was used to predict erosion rate and formation material because neural networks are effective at predicting values with high accuracy^49^. GMDH was first introduced by Ivakhnenko as a polynomial neural network (PNN). It consists of a set of neurons formed by linking pairs through a third-order polynomial. A network with quadratic polynomial composition obtained from all neurons describes the approximate function *f* with an output $\hat{y}_{i}$ for a set of inputs, *X* = (*x*_*1*_*, x*_*2*_*,* … *x*_*n*_) with the least error compared to the actual output *y*. Therefore, for *M* laboratory data including *n* inputs and one output, the actual results are determined (Eq. 3):

3 $$y_{i} = f\left( {x_{i1} + x_{i2} + x_{i3} + ... + x_{in} } \right)\left( {i = 1,2,...,M} \right)$$

To find a network that predicts the output value $\hat{y}_{i}$ for each input vector *X* (Eq. 4):

4 $$\hat{y}_{i} = f\left( {x_{i1} + x_{i2} + x_{i3} + ... + x_{in} } \right)\left( {i = 1,2,...,M} \right)$$

so that it minimizes the mean square error (MSE) between the actual values and the predictions (Eq. 5):

5 $$MSE = \frac{{\sum\limits_{i = 1}^{M} {\left( {\hat{y}_{i} - y_{i} } \right)^{2} } }}{M} \to \min$$

The general form of the connection between the input and output variables can be expressed using the polynomial function (Eq. 6):

6 $$y = a_{0} + \sum\limits_{i = 1}^{n} {a_{i} } x_{i} + \sum\limits_{i = 1}^{n} {\sum\limits_{j = 1}^{n} {a_{ij} } x_{i} x_{j} } + \sum\limits_{i = 1}^{n} {\sum\limits_{j = 1}^{n} {\sum\limits_{k = 1}^{n} {a_{ijk} } x_{i} x_{j} x_{k} } } + ....$$

which is called the Polynomial of Ivakhnenko. In many applications, the quadratic and two variables of this polynomial is (Eq. 7):

7 $$\hat{y} = G(x_{i} + x_{j} ) = a_{0} + a_{1} x_{i} + a_{2} x_{j} + a_{3} x^{2}_{i} + a_{4} x_{j}^{2} + a_{5} x_{i} x_{j}$$

where *a*_*i*_ is the difference between the actual output (*y*) and the calculated values ($\hat{y}_{i}$) for each pair of input variables *x*_*i*_ and *x*_*j*_. A set of polynomials is constructed to determine all unknown coefficients using the least-squares method.

#### Validation model

The mean squared error (MAE), root mean squared error (RMSE) and correlation coefition (R) were used to validat the model (Eq. 8–10)^50^.

8 $$RMSE = \sqrt {\frac{{\sum\limits_{n = 1}^{N} {(Err_{n} )^{2} } }}{N}}$$

9 $$Err_{n} = (Target_{n} - Output_{n} )$$

where $Target_{n}$ is the $n^{th}$ data point to be estimated. While $Output_{n}$ represents the forecasted $n^{th}$ data point estimated by an estimator.

10 $$R = \frac{{N(\sum {xy) - (\sum x )(\sum y )} }}{{\sqrt {[N\sum {x^{2} - (\sum x } )^{2} ][N\sum {y^{2} - (\sum y )^{2} ]} } }}$$

where *N* represents the number of sample points. In addition, *x* and *y* represent output and target points, respectively.

Finally, the proposed framework is prepared in Eq. (11).

11 $$MSE = \left( {1/n} \right) \times \Sigma \left( {actual{-}forecast} \right)^{2}$$

where *n* is number of items, Actual is original or observed y-value, and Forecast is y-value from regression.

All of these statistical analyses were performed in the Statistical Package for the Social Sciences (SPSS) software V. 22, Matlab V. R2017, and ArcGIS V.10.7.1.

## Results and discussion

In this section, firstly, the extracting morphometric characteristics for each of the alluvial fans are descripted in “Morphometric characteristics” section. Then, the classification of alluvial fans using morphometric characteristics is explained in “Classification of alluvial fans using morphometric characteristics” section. Results of selecting important morphometric characteristics using the feature selection algorithm are descripted in “Selecting important morphometric characteristics using the feature selection algorithm” section. Finally, prediction of erosion based on morphometric features using GMDH algorithm are provided in “Prediction of erosion based on morphometric features using GMDH algorithm” section.

### Morphometric characteristics

The four alluvial fans extracted using the semi-automatic method were analyzed and the morphometric characteristics of each were compiled (Table 1). The results indicate that the smallest fan area is in basin 2 (Fan area (*A*_*f*_) = 0.42 km^2^) and the largest is in basin 1 (*A*_*f*_ = 38.63 km^2^). The longest alluvial fan (Fan length (*L*_*f*_) = 9.59 km) is in basin 1 and the shortest is in basin 2 (*L*_*f*_ = 0.74 km). Basin 4 has the lowest minimum fan height (Fan minimum height (*H*_*min-f*_) = 154 m) and the greatest as well (Fan maximum height (*H*_*max-f*_) = 1538 m). The maximum relief ratio is in basin 1 (Fan Relief ratio (*R*_*rf*_) = 1,724.93). And basin 1 has the highest slope (49.5°). The alluvial fan in basin 1 has the greatest radius (9 km). The highest sweep angle (*α)* is also in basin 1 (86°) and the lowest is in basin 2 (16°). Drainage basin shape (*BS*) values in basin 1 (0.82) have the lowest value and basin 4 (3.47) has the highest value. Compactness Coefficient (*C*_*irb*_) also has the lowest value in basin 2 (0.46) and the highest value in basin 1 (6.91).

**Table 1:** Statistical characteristics of morphometric parameters in the study area.

Name	*A*_f_	*P*_f_	*L*_f_	*H*_min_*_f*	*H*_max_*_f*	*ΔH*_f_	*R*_r_*f* _L	*β*_f_	R_f_	Erosion	*α*	BS	*Cirb*	V_f_	*P*_b_	*A*_b_	*H*_min_*_b*	*H*_max_*_b*	*ΔH*_b_	*L*_c_	*L*_b_	*β*_b_	*D*_d_	*Mel*
Basin 1																								
Minimum	0.75	3.57	1.43	985	1036	1010.5	120.8	5.35	0.49	3	31	0.82	0.64	0.11	2.74	0.46	1022	1049	4	0.63	1.03	20.5	141.5	0.04
Maximum	38.63	26.84	9.59	10,990	1392	6054.5	1724.93	49.5	9	7	86	3.9	6.91	11.6	33.66	62.52	1411	2077	813	11.77	13.56	69.5	1135	93.3575
Average	7.09	10.07	3.91	1459.88	1217.27	1336.13	436.6	29.65	2.28	6.04	62.18	1.93	2.48	2.03	12.16	12.46	1216.32	1543.32	329.21	3.77	4.73	44.79	754.79	15.47
STDEV	7.68	5.6	2.2	1348.05	100.91	672.1	263.81	8.28	1.42	1.03	12.54	0.62	1.66	2.41	7.31	13.91	112.65	307.47	232.46	2.7	3.02	10.84	257.82	18.47
Basin 2																								
Minimum	0.42	2.43	0.74	663	673	668	318.27	0.89	0.8	4	16	0.58	0.44	0.04	3.93	1.1	665	732	719	0.86	1.24	2.69	495.5	123.875
Maximum	6.43	10.12	2.19	682	725	698	915.43	3.43	3.8	7	65	1	5.74	0.53	15.62	13.37	735	1123	928	1.93	3.57	25.5	1042	260.5
Average	2.13	5.02	1.27	672.23	698.54	684.96	639.68	2.17	1.83	6.15	38	0.76	1.96	0.25	7.79	4.78	694.54	951.31	824.42	1.42	2.29	16.53	712.35	178.0875
STDEV	2.28	2.88	0.56	6.28	18.44	10.68	234.59	0.83	1.08	1.03	17.65	0.13	1.83	0.18	4.09	4.45	24.19	133.27	70.24	0.37	0.77	7.1	221.33	55.3325
Basin 3																								
Minimum	0.34	2.68	1.06	154	198	176	52.44	1.18	0.62	7	26	0.94	0.47	0.13	4.91	1.66	176	310	257	1.4	1.9	5.7	674.5	168.625
Maximum	12.08	12.71	4.14	213	258	226	174.04	3.1	3.9	7	82	1.88	6.82	1.24	21.38	32.42	279	548	413.5	5.37	6.76	17.75	1417.5	354.375
Average	3.08	6.16	2.17	175.38	217.92	195.92	105.62	1.94	1.66	7	60.62	1.45	1.89	0.6	10.73	10.22	221.62	403.54	313.65	2.7	3.59	10.85	1101.19	275.2975
STDEV	3.91	3.12	0.98	20.15	22.81	18.95	38.59	0.67	1.05	0	19.3	0.34	2.14	0.41	5.34	10.54	33.1	85.56	55.25	1.39	1.59	4.67	224.11	56.0275
Basin 4																								
Minimum	1.89	5.62	1.88	1283	1385	1334	271.07	1.65	1.2	7	32	0.83	0.96	0.47	9.81	5.57	1411	1722	1578.5	2	3.77	11.95	904.5	226.125
Maximum	12.53	16.11	5.22	1367	1538	1452.5	740.96	7.9	5.5	7	79	3.47	5.02	6.08	22.58	35.58	1773	2607	2055	7.7	8.42	21.65	1415.5	353.875
Average	6.28	10.19	3.64	1328.57	1461	1394.79	431.83	4.54	2.69	7	55.93	1.69	2.65	1.93	14.3	14.46	1523.5	2155.86	1830.89	3.94	5.6	17.63	1150.39	287.5975
STDEV	3.58	3.37	1.11	30.9	50	39.23	166.8	2.37	1.38	0	14.36	0.85	1.27	1.83	4.27	9.7	112.38	314.36	173.35	2.08	1.61	3.27	184.63	46.1575

Fan volume (*V*_*f*_) is the highest in basin 1 (11.6) and the lowest in basin 2 (0.04). In terms of the morphometric characteristics of the recharged watershed of each alluvial fan, the highest and lowest basin area (*A*_*b*_) in basin 1 are 62.52 and 0.46 km^2^. The maximum height of basin (*H*_*max_b*_) is in basin 3 (176 m) and the basin minimum height (*H*_*min_b*_) is in basin 4 (1722 m). The maximum and minimum main channel length (*L*_*c*_) are in basin 1 (11.77) and (0.63). And basin 1 has the highest basin length (*L*_*b*_) value (13.5). The maximum and minimum drainage density (*Dd*) values are in basin 3 and 1. The steepest slope (*SS*) is in basin 1 (69.5°) and the gentlest slope us in basin 2 (2.69°). The maximum and minimum Melton’s number (*Mel*) values are in basins 1 and 3. The least erosion is occurring in basin 1.

**Figure 5:**
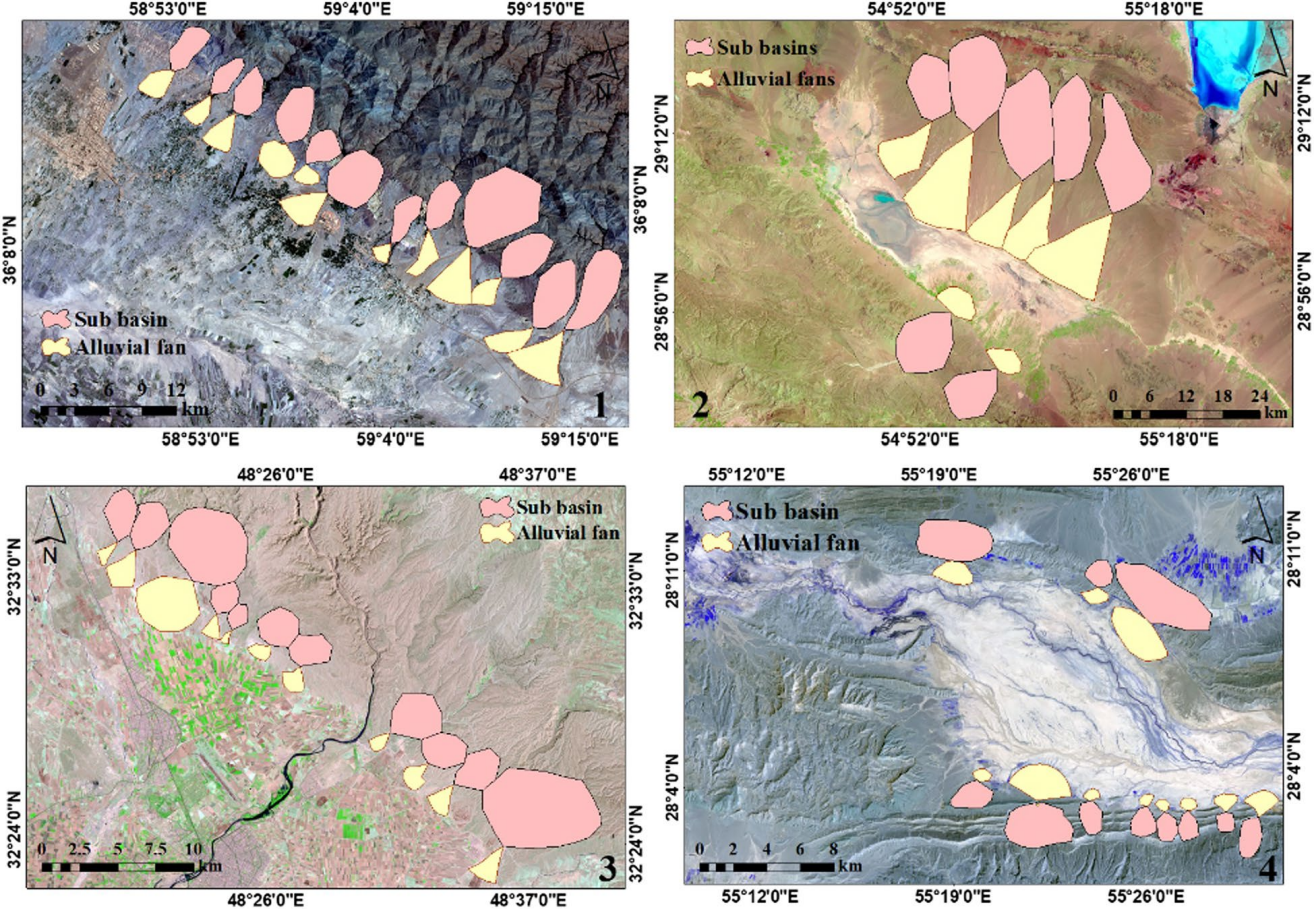
Position of alluvial fans in the study area.

The 4 basins have different geological genera (Fig. 5). Basin 1, located in the Central Desert watershed, has four types of formations: Qft1 (high-level piedmont fan and valley terrace deposits), Qft2 (low-level pediment fan and valley terrace deposits), Plms (marl, shale, sandstone, and conglomerate), and Eavt (andesitic volcanic tuff). Qft1 and Eavt are moderately erodible and Qft2 and Plms are highly erodible. Basin 2, located in the Mehran watershed, has OMR (red, grey, and green silty marls interbedded with subordinate silty limestone and minor sandstone ribs) and Eoas-ja (undivided Asmari and Jahrum formations, regardless of the disconformity separates them) formations, and both of these are moderately erodible. Basin 3, located in the Karun watershed, is mainly Plbk (alternating hard deposits of consolidated, massive, features forming conglomerates and low-weathering cross-bedded sandstone) that is moderately erodible^51^. And the geology of basin 4, located in the Mehran watershed, comprises the MUR (red marl, sandstone, and conglomerate) and is highly erodible^52^.

**Figure 6:**
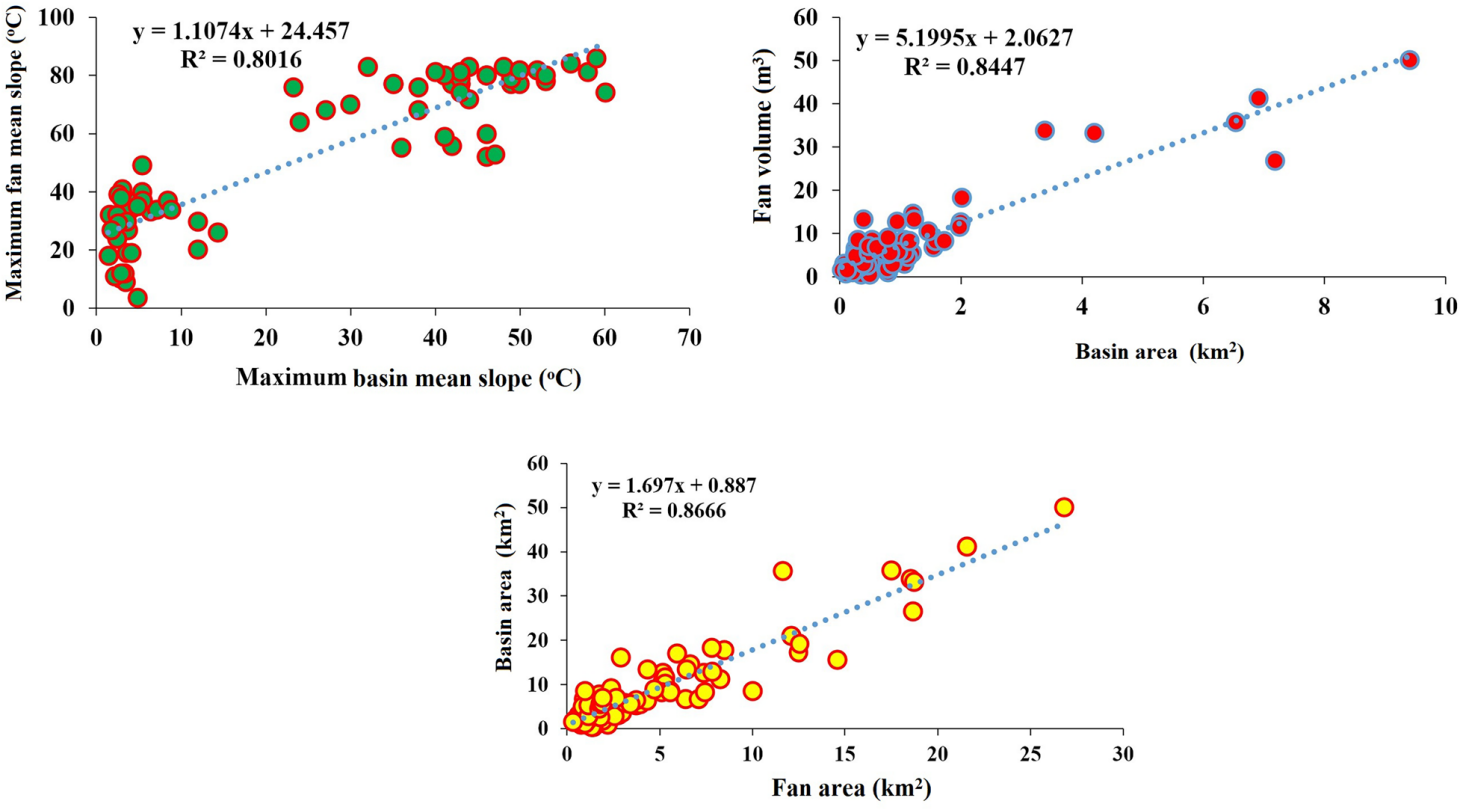
Relationship between *A*_*f*_*-A*_*b*_, Maximum β_b_- Maximum β_f_, *A*_*b*_*-V*_*f*_*.*

The relationships between the alluvial fan area and the upstream catchment area (Fig. 6) are positive and significant (R^2^ = 0.86). Watershed slope and alluvial fan slope (R^2^ = 0.80) and alluvial fan volume with alluvial fan area (R^2^ = 0.84) are highly correlated pairs.

### Classification of alluvial fans using morphometric characteristics

SOM algorithm was applied to the 25 morphometries properties: *A*_*f*_, Fan perimeter (*P*_*f*_), *L*_*f*_, Fan minimum height (*H*_*min_f*_), Fan relief (*ΔH*_*f*_), Fan Relief ratio (Rr_f_), Fan mean slope (β_f_), Upper fan slope (*β*_*upp_f*_), *α*, Cirb (*CC*), *BS*, *V*_*f*_, *A*_*b*_, Basin perimeter (*P*_*b*_), *L*_*b*_, *L*_*c*_, *H*_*min_b*_, *H*_*max_b*_, Basin relief (*ΔH*_*b*_), Basin Relief ratio (*Rr*_*b*_), Basin mean slope (*β*_*b*_), *Dd*, *Mel* and the results were mapped (Fig. S3). Each SOM matrix map represents an index value obtained after dimension reduction, as marked by shades of blue to red^53^.

High-value neurons are red. Low-value neurons are blue. SOM maps enable visual comparison of the parameters according to the color gradients. Parameters *L*_*f*_*, H*_*min_f*_*, and β*_*min-f*_ have the same color gradient changes, showing that these 3 parameters are positively and strongly correlated. Similarly, the parameters *H*_*max_f*_ and *A*_*f*_ are correlated.

**Figure 7:**
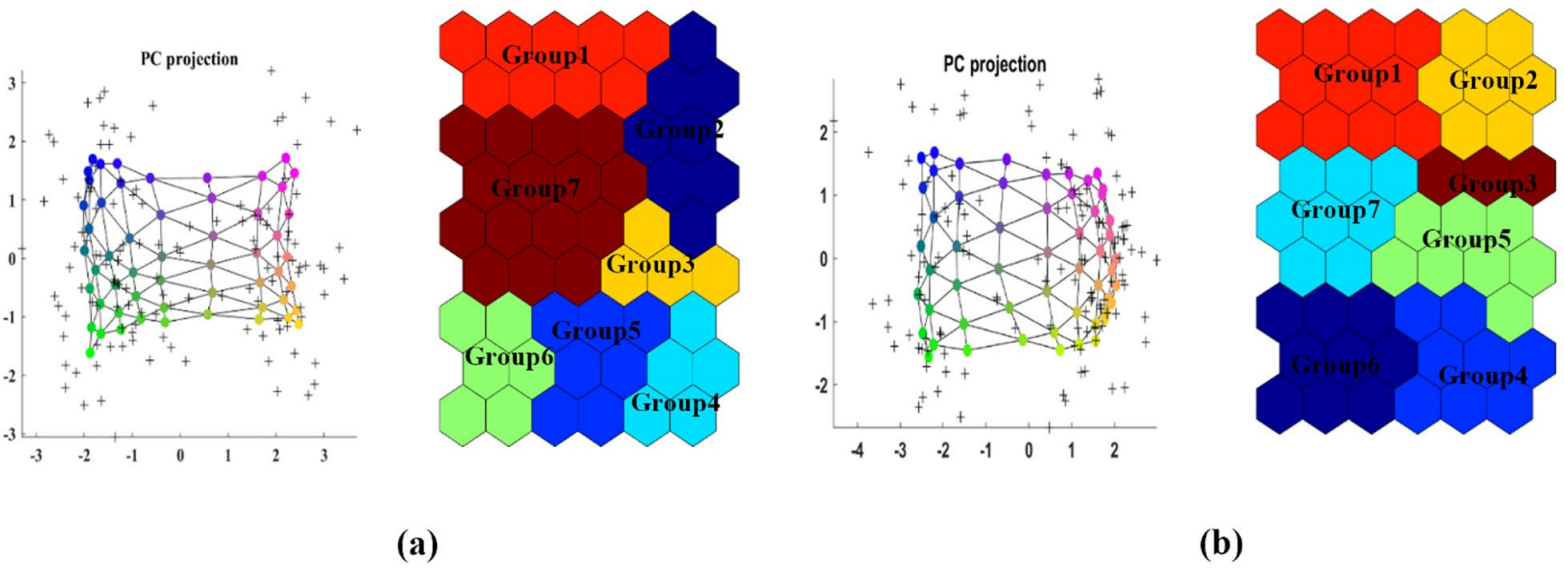
Visualizations of the clusters obtained from the classification of the morphometric parameters using SOM (**a**) lithology, (**b**) erosion.

The clustered distributions of alluvial fan morphometric parameters and the upstream watershed were also examined (Fig. 7). Group 1 contains *P*_*f*_ (upper left corner), group 2 contains *P*_*b*_ (upper right corner), group 3 contains *β*_*max-f*_ (center right), group 4 contains *L*_*f*_ (lower right corner), group 5 contains *β*_*min-f*_, *α*, and CC (bottom center), the group 6 contains *H*_*max_f*_, *BS*, and *Dd* (lower left corner), and group 7 includes *H*_*max_b*_, and *β*_*max-b*_. Groups 1 and 7 have the greatest impact on the formation of the study area.

The 7 clusters are also related to geological formations: group 1 includes Plms, Qft1, Ogr, and Qft2; group 2 includes Plms and Qft2; group 3 includes Qft1and Ogr; group 4 includes the Eoas-ja formation; group 5 includes Mur, Omr, and Eoas-ja; group 6 includes Mur, Plbk, Plc, and Eav; and group 7 includes Qft2, Qft1, and Ogr. Erosion (Fig. S3) caused the same slope changes with *L*_*f*_*,H*_*min_f*_*, H*_*max_f*_ and* β*_*min-*f_ positively correlated. P_f_ and CC are highly correlated. The morphometric feature distribution for erosion (Fig. 7) reveals that group 1 includes P_f_ and Rr_f_; group 2 includes *L*_*c*_; group 3 includes *H*_*max_f*_; group 4 includes *H*_*max_b*_; group 5 includes *L*_*f*_*, H*_*max_f*_*, P*_*b*_, and *H*_*min_b*_; group 6 includes *H*_*max_f*_ and *β*_*max-b*_; and group 7 includes α. Groups 1 and 3 are in erosion class 3; group 2 is in erosion class 4; groups 5 and 6 are in erosion class 7; and group 7 is in erosion class 5. Visualization of the relationships between morphometrics properties and erosion and lithology values is shown in Fig. S3.

### Selecting important morphometric characteristics using the feature selection algorithm

The feature selection algorithm was used to select the most important morphometric parameters. The data were divided into two sets to create the models: 70% (about 77 samples) of the data were used for training and 30% (about 33 samples) for testing^54^. The models were CFC + Greedy, CFS + Best first, CFS + Genetic search, and CFS + Random search. R^2^ was used to determine the accuracy of the predicted values relative to the actual values and thus was used for validation (Table 3). The results indicate that the CFC + Greedy model is a more accurate model for predicting erosion using *H*_*min-f*_*, H*_*max-f*_*, β*_*min-f*_, and *L*_*f*_. The R^2^ values reveal that the CFS + Genetic search model was the most accurate (R^2^ = 0.95) to predict fan formation with *A*_*b*_*, A*_*f*_*, H*_*max-f*_*,* and *CC*. This algorithm is used primarily to reduce the number of variables (from the 25 morphometric characteristics) and to identify the variables most important for the next steps.

**Figure 8:**
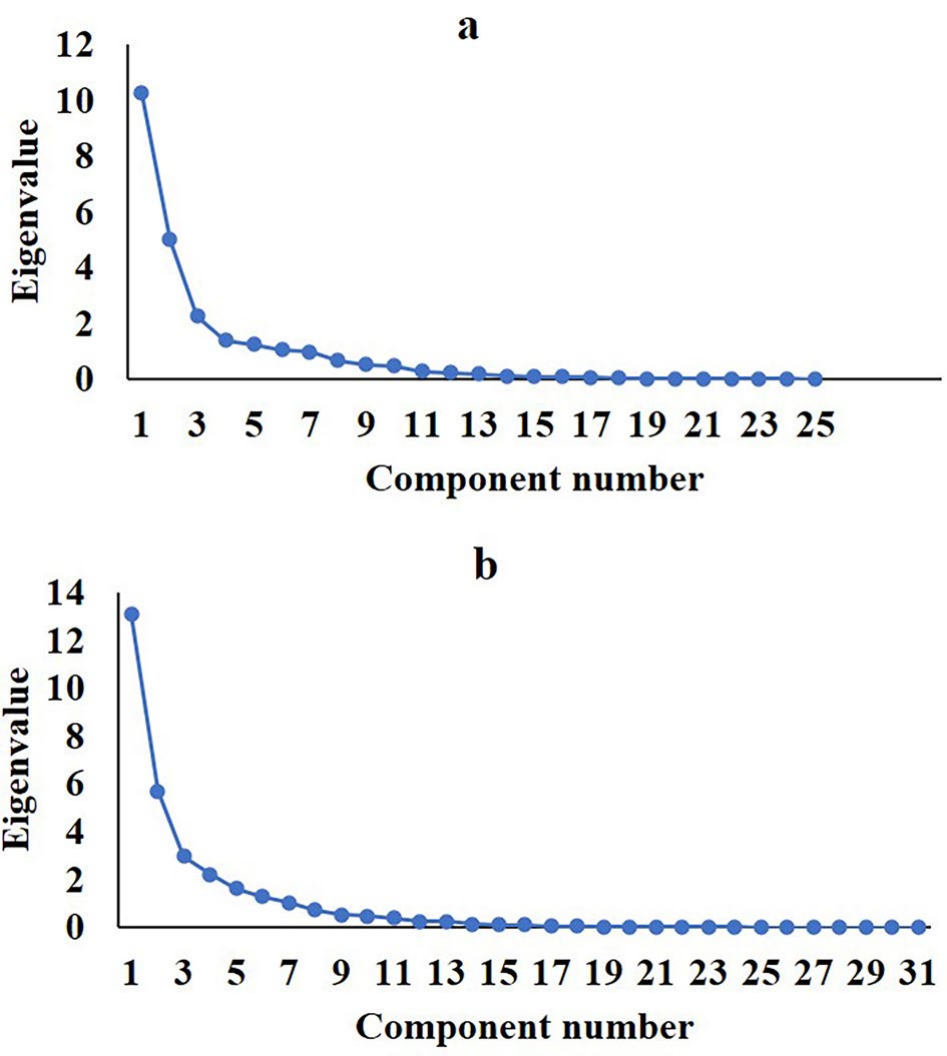
Number of components of erosion (**a**), and lithology (**b**).

Inaddition, the most relevant variables for determining soil erosion rate and formation characteristics were determined using PCA. According to the results, soil erosion has three components. The first and second components represent 87% of the specific values. In this method, 64% of the changes are seen in the first component, which includes *A*_*f*_*, P*_*f*_*, L*_*f*_*, V*_*f*_*, P*_*b*_*, A*_*b*_*, L*_*c*_*,* and *L*_*b*_. According to the type of formation, the first and second components account for 89% of the specific value. The first component includes *A*_*f*_, *P*_*f*_, *L*_*f*_, *α, P*_*b*_, *A*_*b*_, *H*_*max_b*_, *C*_*irb*_*,* and *Altitude* / *L*_*b*_, which represent 53% of the changes (Fig. 8 and Tables 2 and 3).

**Table 2:** Average percentage of changes expressed for erosion.

Variable	Component		
	1	2	3
Fan area (*A*_*f*_)	.902	− .198	− .086
Fan perimeter (*P*_*f*_)	.966	− .125	− .072
Fan length *(L*_*f*_)	.959	.021	− .122
Fan minimum height (*H*_*min-f*_)	.155	.333	.361
Fan maximum height (*H*_*max-f*_)	.535	.634	.479
Altitude/*L*_*f*_	− .503	.345	.509
Minimum β_f_	.176	.760	− .172
Maximum β_f_	.436	.695	− .304
Fan Relief ratio (Rr_f_)	.770	− .353	.060
Sweep angle (*α)*	.153	.466	− .344
Drainage basin shape (*BS*)	.334	.534	− .249
Compactness Coefficient (*C*_*irb*_)	.680	− .261	.022
Fan volume (V_f_)	.862	− .103	− .111
Basin perimeter (*P*_*b*_)	.910	− .306	− .019
basin area (*A*_*b*_)	.876	− .294	− .087
Minimum height of basin (*H*_*max_b*_)	.540	.619	.500
Maximum height of basin (*H*_*max_b*_)	.640	.439	.555
Melton’s number (*Mel*)	− .371	.393	− .079
*C*_*irb*_	− .270	− .440	− .035
Altitude/length sub basin	.166	− .331	.807
Channel length (*L*_*c*_)	.908	− .165	− .087
basin length (*L*_*b*_)	.921	− .186	.003
Maximum slope of sub basin	.459	.735	− .176
Minimum slope of sub basin	.398	.597	− .158
Drainage density (*Dd*)	.543	− .622	.103

**Table 3:** Average percentage of changes expressed for lithology.

Variable	Component		
	1	2	3
Fan area (*A*_*f*_)	.883	− .301	.068
Fan perimeter (*P*_*f*_)	.929	− .290	− .027
Fan length *(L*_*f*_)	.909	− .255	− .021
Fan minimum height (*H*_*min*_-_*f*_)	.329	.117	.858
Fan maximum height (*H*_*max*_-_*f*_)	.402	.644	− .058
Altitude/*L*_*f*_	− .053	.415	.805
Minimum β_f_	.298	.626	.092
Maximum β_f_	.122	.707	.043
Fan Relief ratio (Rr_f_)	.363	.345	.168
Sweep angle (*α)*	.847	− .236	.043
Drainage basin shape (*BS*)	.169	.527	.228
Compactness Coefficient (*C*_*irb*_)	.285	.258	.436
Fan volume (V_f_)	.726	− .360	.073
Basin perimeter (*P*_*b*_)	.861	− .241	.060
basin area (*A*_*b*_)	.921	− .297	− .008
Minimum height of basin (*H*_*max_b*_)	.880	− .314	.052
Maximum height of basin (*H*_*max_b*_)	.438	.682	− .086
Melton’s number (*Mel*)	.720	.542	− .323
*C*_*irb*_	.887	− .326	.053
Altitude/*L*_*b*_	.893	− .310	.035
Channel length (*L*_*c*_)	.526	.632	− .326
basin length (*L*_*b*_)	.465	.560	− .162
Maximum slope of sub basin (β_f_)	.688	.007	− .043
Minimum slope of sub basin (β_f_)	.792	− .240	− .075
drainage density (*Dd*)	.792	− .240	− .075

### Prediction of erosion based on morphometric features using GMDH algorithm

Data selected with the feature selection algorithm were used as input to predict erosion rates and formation types by GMDH algorithm. These data were divided into two categories for educating (70%) and testing (30%) the algorithm^55^. In the research, the GMDH structure design was used as an initial population of 100, Crossover probability of 0.84, and mutation probability of 0.15 in 200 generations (replication).

Table 4 shows the polynomial functions developed with the GMDH algorithm for predicting erosion rates, and formation types. Several polynomial equations have been used to predict these parameters in the algorithm. The results show that morphometric parameters (*A*_*b*_, *A*_*f*_, *H*_*max-f*_, and *CC*) are effective in predicting formation types. For predicting erosion rates, morphometric parameters such as *H*_*min-f*_, *H*_*max-f*_,* β*_*min-f*_, and *L*_*f*_ are effective.

**Table 4:** Polynomial equations for erosion rates, formation types using GMDH method.

Formation types	$Y_{1} = 6.62 - 0.006A_{f} + 0.017A_{b}$
	$$Y_{2} = 4.1 + 0.023H_{{\max_{f} }} - 10.12CC$$
	$$Y_{3} = 2.1 + 0.13Y_{2} + 0.12CC$$
	$$Formation \, types = 5.2 + 0.12Y_{1}^{2} + 0.033Y_{2} - 0.46Y_{3}^{2}$$
Erosion rates	$Y_{1} = 4.24 + 0.03H_{{\min_{f} }} - 0.02\beta_{\min }$
	$$Y_{2} = 3.8 + 0.21H_{{max_{f} }} + 0.29L_{f}$$
	$$Y_{3} = 5.3 + 0.37H_{{max_{f} }} + Y_{1}$$
	$$Erosion \, rates = 2.43 + 0.8Y_{1} \times 0.6Y_{2} - 0.12Y_{3}^{2}$$

**Figure 9:**
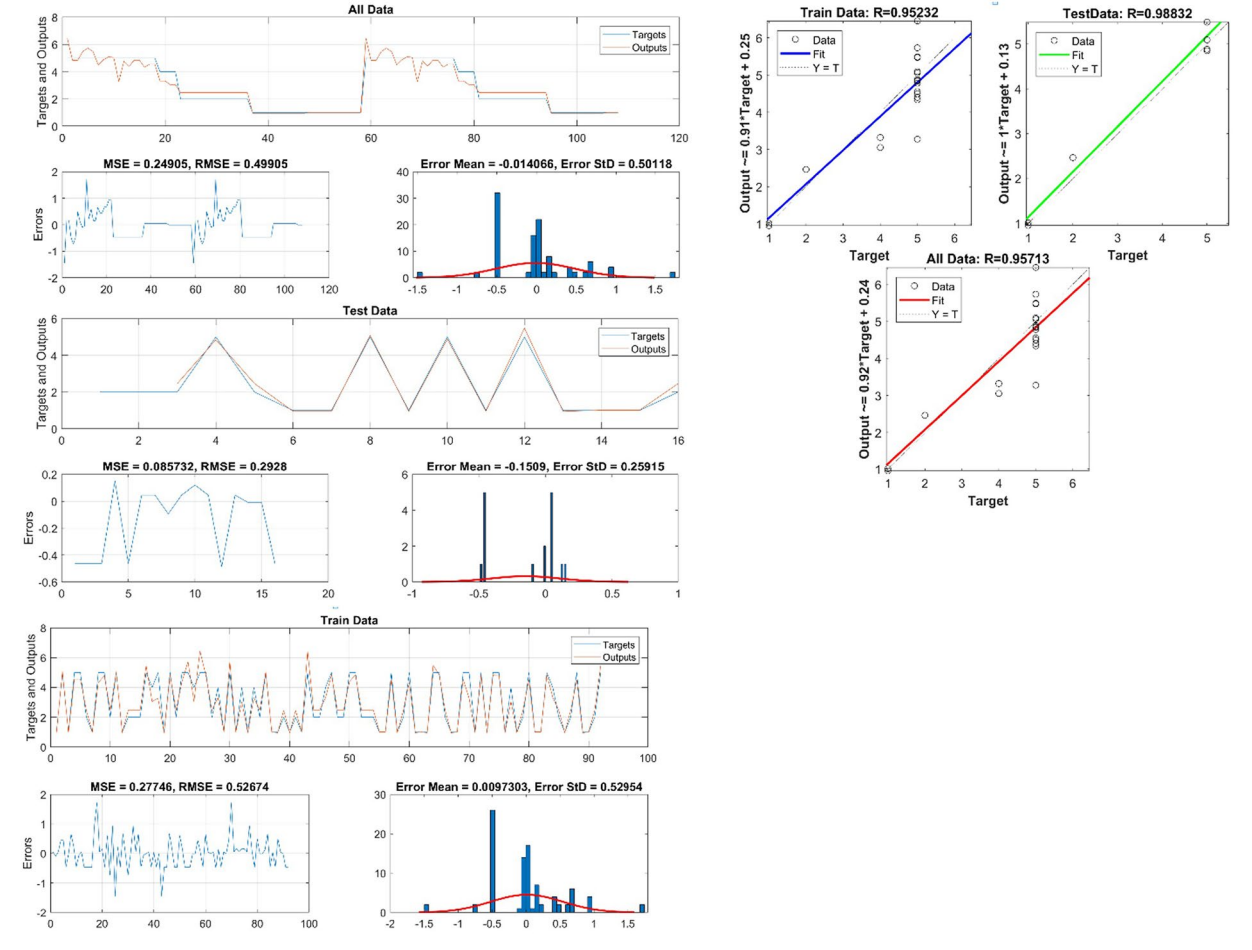
Comparison between input data and predicted data by GDMH for lithology.

**Figure 10:**
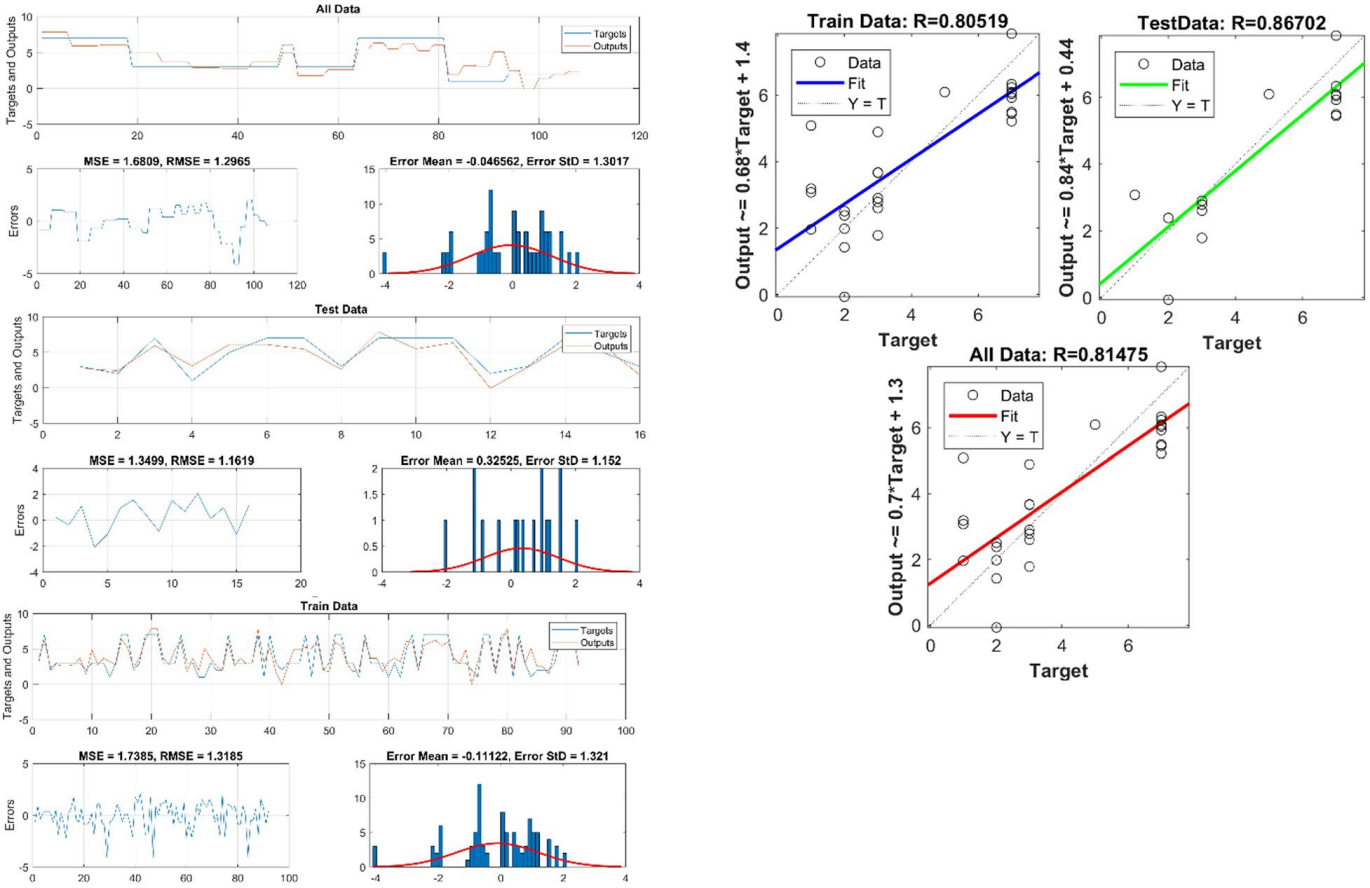
Comparison between input data and predicted data by GDMH for erosion.

The input and output systems of GMDH neural networks showed 6 hidden layers and the best combination was RMSE_erosion_ = 0.57 and RMSE_lithology_ = 0.42 than 2, 3, 4, 5, 7 and 8 hidden layers (Table 5). As the number of hidden layers increased, the RMSE decreased, and the accuracy of the model to predict erosion and lithology increased. Therefore, with the number of hidden layers, 6 predictions were made by the GMDH algorithm. The testing and training data were graphed for lithology (Fig. 9) and erosion (Fig. 10). The R^2^_test_ = 0.98, R^2^_train_ = 0.95, and R^2^_all_ = 0.95, indicating that the GMDH model very accurately predicted lithologies. And R^2^_test_ = 0.86, R^2^_train_ = 0.8, and R^2^_all_ = 0.81, indicating that the GMDH model accurately predicted erosion rates. GMDH, therefore, is shown to provide highly accurate forecasting.

**Table 5:** RMSE values for the number of different hidden layers.

Step	Hidden layers	RMSE	Step	Hidden layers	RMSE
Erosion			Lithology		
1	8	0.61	1	8	0.43
2	7	0.59	2	7	0.43
3	6	0.57	3	6	0.42
4	5	0.69	4	5	0.54
5	4	0.72	5	4	0.68
6	3	0.87	6	3	0.83
7	2	0.93	7	2	0.89

To predict erosion rates, and formation types, Figs. 9, 10 show correlation and error data for testing, training, and total data. These figures show that R^2^ has high values, which illustrate the model's high accuracy in predicting the erosion rates, and formation types. These parameters can be predicted using formulas on the Y axis. As can also be seen in Figs. 9, 10, the error values have been low, which indicates a high accuracy forecast model, with low values of RMSE, MSE, Error mean, and Error STD for all parameters of erosion rates, formation types.

The receiver operating characteristic (ROC) curve was also used in this study to assess the accuracy of the results. To test the validity of the prediction, the actual erosion and formation values were compared with those predicted at 20 points in the study area, as shown in Table 6. Based on the results, the GMDH method is very accurate at predicting erosion and formation types.

**Table 6:** Area Under the Curve of built models.

Models	Area	Standard error	Asymptotic significant	Asymptotic 95% confidence interval	
				Lower bound	Upper bound
Lithology	0.9879	0.05148	0.00396	0.887	1.08881
Erosion	0.83733	0.18027	0.04633	0.48401	1.19066

Based on the results, only a few morphometric parameters are required to investigate alluvial fan geological characteristics and erosion rates. The results showed that geology is one of the most important parameters affecting the morphology of alluvial fans^25^. The geological characteristics of the watershed control the rates of erosion and weathering, and the morphometry of alluvial fans in an area^56^. This study showed that basins 1 and 2 are the most erosion-resistant and weathering, most likely due to the younger sediments. Basins 3 and 4, on the other hand, are more erodible and this affects alluvial fan morphometry, yielding fans that have excellent soil for agriculture. Basins 1 and 2 have elongated and angled alluvial fans due to the low erosion rates in these areas. Based on these findings, it can be concluded that basins 1 and 2 are more suitable as sites for extracting sediments for use as building materials as the sediments are less weathered. Basins 3 and 4, on the other hand, are more erodible and are better suited to agricultural uses. Therefore, using the morphometric characteristics of alluvial fans, erodibility and lithologies can be determined^25^. Using neural network models, active processes in the region can be predicted based on morphometric features^57^.

The studies show that ANNs have been used in many fields. A large number of systems have been discovered with ANN, a network of interconnected neurons that can be associated with different functions^57^. The GMDH neural network is a special type of ANN that uses a search and error method to find the optimal network. It creates topologies with varying numbers of hidden neurons^49^.

Studies show that the alluvial cone surface in many areas is dedicated to agricultural fields, gardens, rural places, and communication networks. On the other hand, determining the rate of erosion in alluvial cones determines the activity level of the prevailing processes in the region. Therefore, predicting the amount of erosion in alluvial cones in relation to the shape of alluvial cones helps a lot in the management of these areas. Javanbakht et al. showed that there is a direct relationship between alluvial cone shape and erosion rate. So that by using the form, it is possible to predict the ruling processes in the region for the management of these regions which is consistent with the results of this study.

Also, studies show that the type of formation plays a significant effect on the resistance of the formation against erosion and finally the appearance of different shapes in the study area. By identifying and predicting the type of formation, it is possible to predict the region's situation in terms of landslides, falls, and waterway density. Golestani et al. Golestani et al.^58^ showed in their studies that it is very important to identify the type of formations in the region to control erosion and the resulting risks, which is consistent with the results of this study.

The researchs show that GMDH does not require advanced planning. The only parameter to adjust is the threshold limit for removing network units. Also, this network can determine effective inputs to solve the problem^59^. In the GMDH network, instead of building estimator models at once, an iterative and incremental algorithm is used, which includes the generation and addition of very simple basic structures (polynomial neurons). In this method, over time, by combining these simple structures, a complex system is formed that has optimal performance. In the GMDH method, unlike other regression methods, in addition to the gradual construction of the model, the Natural Selection pattern is used, similar to what is used in evolutionary algorithms^60^. GMDH is an inference algorithm and it can be considered a regression-based method that combines the advantages of both regression methods and neural networks. The evaluation of neural networks is more difficult than the GMDH method because it is difficult to interpret the weights^61^. Unlike GMDH, all input variables without considering their effect on the network structure predict the desired goal.

Among other methods for predicting and checking the amount of erosion and the type of formations, meta-heuristic algorithms can be mentioned, which have more solutions than the methods used in this study and the possibility of optimizing problems in wider dimensions. Therefore, it is suggested to use these methods in future studies to investigate and determine the relationship between forms and processes in watersheds^62^. It is suggested that according to the studies conducted by Mehrabi et al. and Moayedi et al.^27,63^, in subsequent studies, methods such as particle swarm optimization (PSO) and chimp optimization algorithm (ChOA), crow search algorithm (CSA), satin bowerbird optimization (SBO), and water cycle algorithm (WCA) can be used to determine the relationship between forms and processes in watersheds.

## Conclusion

It is concluded that there is a relationship between the morphometrics of the alluvial fan, its recharged watershed, and ongoing processes like erosion. Watershed processes can be predicted using morphometrics. The results show that more elongated alluvial fans experience less erosion and are more suitable sites for mining competent building material. Rounder alluvial fans reflect higher rates of erosion. It has been demonstrated that SOM neural networks can be used to visually investigate the relationships between morphometric alluvial fan features and upstream watershed characteristics. The results also showed that the GMDH neural network can be used to predict erosion and lithology with high accuracy from morphometric features. the processes at work in watersheds with alluvial fans can be determined and predicted with high accuracy using the semi-automated method using neural networks and morphometric data. Due to the close relationship between form and process, it is suggested that methods such as the long short-term memory (LSTM) neural network method be used to predict processes and then forms in the future so that more accurate management can be done. In future studies, satellite images with a higher resolution should also be used to extract forms.

## Supplementary Information

Supplementary Information 1.

Supplementary Information 2.

## Data Availability

The datasets used and generation during the current study can be downloaded from the following link: https://figshare.com/articles/dataset/data_SR_zip/21329049.
