# Author Correction: Using machine learning to predict processes and morphometric features of watershed

**DOI:** 10.1038/s41598-023-37923-2

**Published:** 2023-07-03

**Authors:** Marzieh Mokarram, Hamid Reza Pourghasemi, John P. Tiefenbacher

**Affiliations:** 1 Department of Geography, Faculty of Economics, Management and Social Sciences, Shiraz University, Shiraz, Iran; 2 Department of Soil Science, College of Agriculture, Shiraz University, Shiraz, Iran; 3 Department of Geography, Texas State University, San Marcos, TX USA

Correction to: *Scientific Reports* 10.1038/s41598-023-35634-2, published online 25 May 2023

The original version of this Article contained an error in Affiliation 1, which was incorrectly given as ‘Department of Geography, Faculty of Economics, Management and Social Sciences, Shiraz University, Shiraz, Darab, 71379-58756, Iran’. The correct affiliation is listed below:

Department of Geography, Faculty of Economics, Management and Social Science, Shiraz University, Shiraz, Iran.

The original Article has been corrected.

