# Supplementary material for: Using machine learning to predict processes and morphometric features of watershed
Source: Sci Rep. 2023 May 25;13:8498. doi: 10.1038/s41598-023-35634-2 (PMC10212989; doi:10.1038/s41598-023-35634-2)
Supplement: Supplementary file 2 — Supplementary Information 2. [file 41598_2023_35634_MOESM2_ESM.docx]

**Supporting Information for**

**Using Machine Learning to Predict Processes and Morphometric Features of Watershed**

**Marzieh Mokarram^1*^ Hamid Reza Pourghasemi^2^ John P. Tiefenbacher^3^**

*^1^ Department of Geography, Faculty of Economics, Management and Social sciences, Shiraz University, Shiraz, Iran, Email:* [*m.mokarram@shirazu.ac.ir*](mailto:m.mokarram@shirazu.ac.ir)

*^2^Department of Natural Resources and Environmental Engineering, College of Agriculture, Shiraz University, Shiraz, Iran, Email:* [*hr.pourghasemi@shirazu.ac.ir*](mailto:hr.pourghasemi@shirazu.ac.ir)

*^3^Department of Geography, Texas State University, San Marcos, TX USA, Email: tief@txstate.edu*

***^*^Corresponding author:*** *Marzieh Mokarram, Tel.: +98-917-8020115; Fax: +987136134479 Address: Shiraz university, Iran, Postal Code: 71379-58756, Email: m.mokarram@shirazu.ac.ir*

**Table S1.** Morphometric parameters characteristics

| Symbol | Variable | Symbol | Variable |
| --- | --- | --- | --- |
| *A*_b_ | Basin area (m^2^) | *D*_d_ | Drainage density (km^−1^) |
| *P*_b_ | Basin perimeter (m) | *Mel* | Melton’s number  *Mel* = *ΔH*_b_/(*A*_b_)0.5 |
| *L*_b_ | Basin Length (m) | *A*_f_ | Fan area ( m^2^) |
| *L*_c_ | Main channel length (m) | *P*_f_ | Fan perimeter (m) |
| *H*_min_*_b* | Basin minimum height (m) | *L*_f_ | Fan length (m) |
| *H*_max_*_b* | Basin maximum height (m) | *H*_min_*_f* | Fan minimum height (m) |
| *ΔH*_b_ | Basin relief (m)  *ΔH*_b_= *H*_max_*__b_* − *H*_min_*__b_* | *ΔH*_f_ | Fan relief (m)  *ΔH*_f_ =  *H*_max_*_f* − *H*_min__*f* |
| *R*_r_*b* | Basin Relief ratio (m) *R*_r_*b* = *ΔH*_b_/*L*_b_ | *R*_r_*f* | Fan Relief ratio  *R*_r_*f* = *ΔH*_f_/*L*_f_ |
| *β*_b_ | Basin mean slope (°) | *β*_f_ | Fan mean slope (°) |
| *BS* | Basin shape | *BL/BW* | BL:Basin length  BW: Basin width |
| *β*_low_*_b* | Lower basin slope (°) | *β*upp*_f* | Upper fan slope (°) |
| *β*_c_ | Main channel slope (°) | *α* | Sweep angle (°) |
| *Cirb* | *Cirb*  $v=A_{b}/(\frac{\piГ2\alpha}{360})$  π is equal to 3.14, Г is the radius of the cone and α is the angle between the two margins of the alluvial fan at the junction of the alluvial fan | *V_f_* | *Fan volume* (m^3^)$V=(\frac{\pi\times r^{2}\times h}{3})\times(\frac{s}{360})$  *π* is the fan radius in meters, *h* is the height difference between the apex and the base of the fan in meters, *S* is the sweeping angle |

**
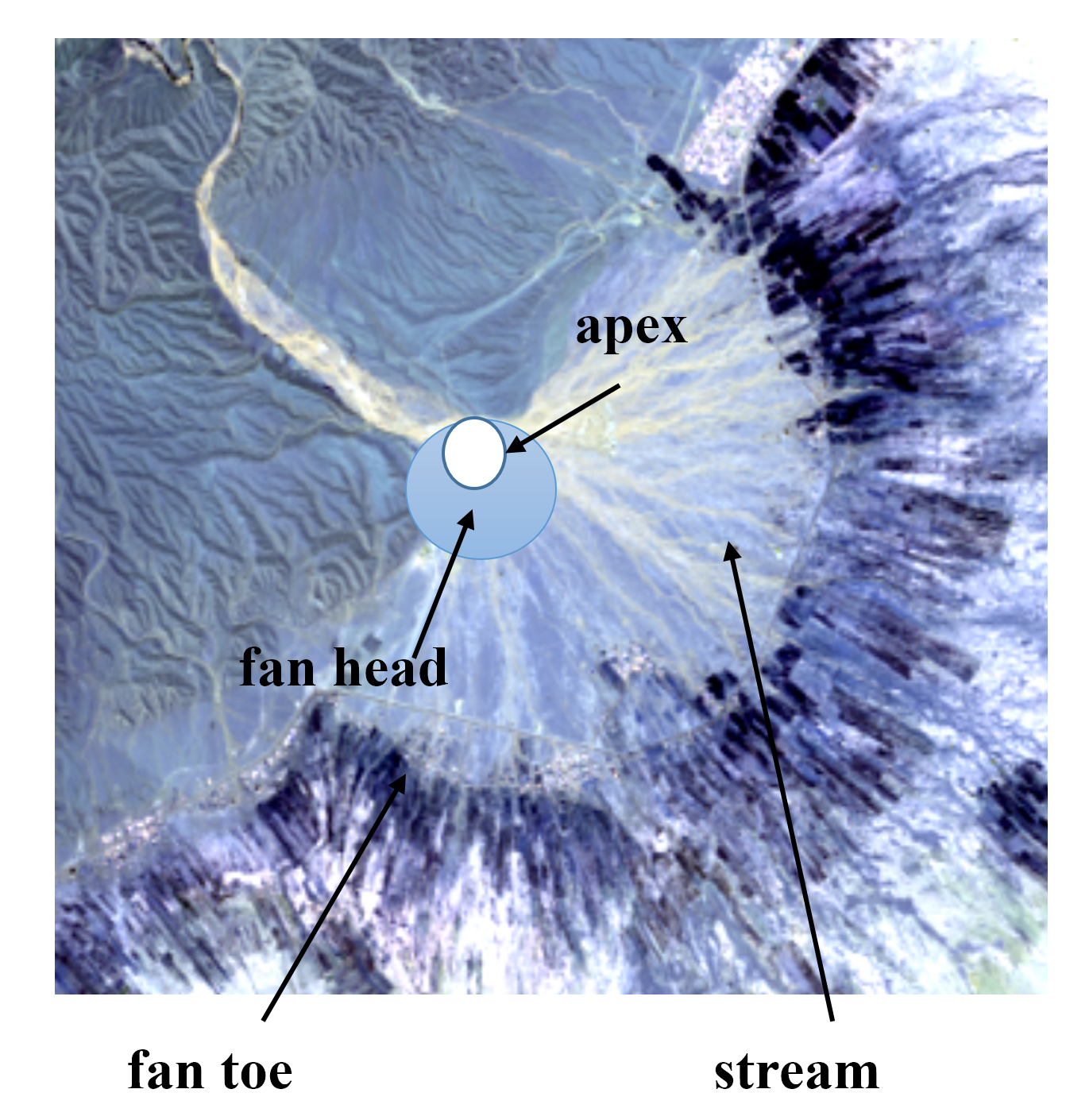
**

**Fig. S1.** The structure of an alluvial fan

**Fig. S2.** Steps of research


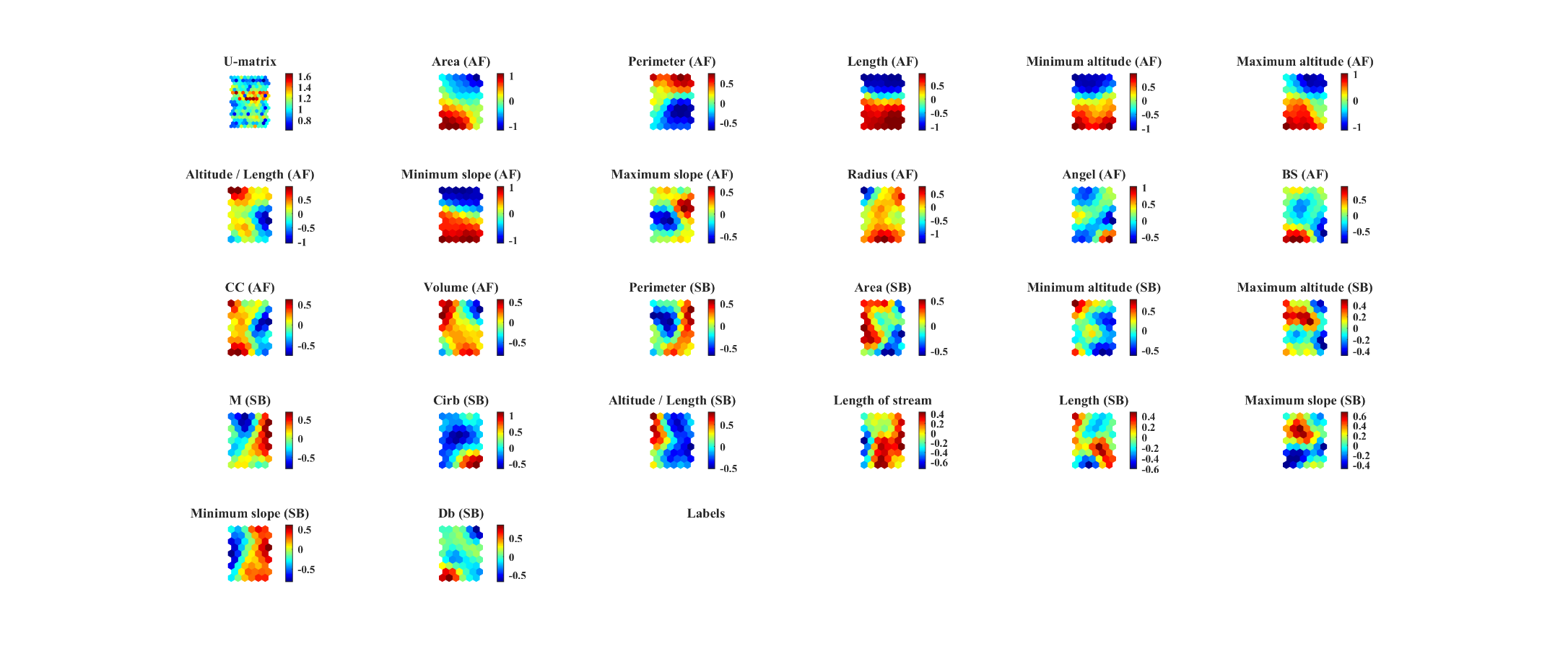

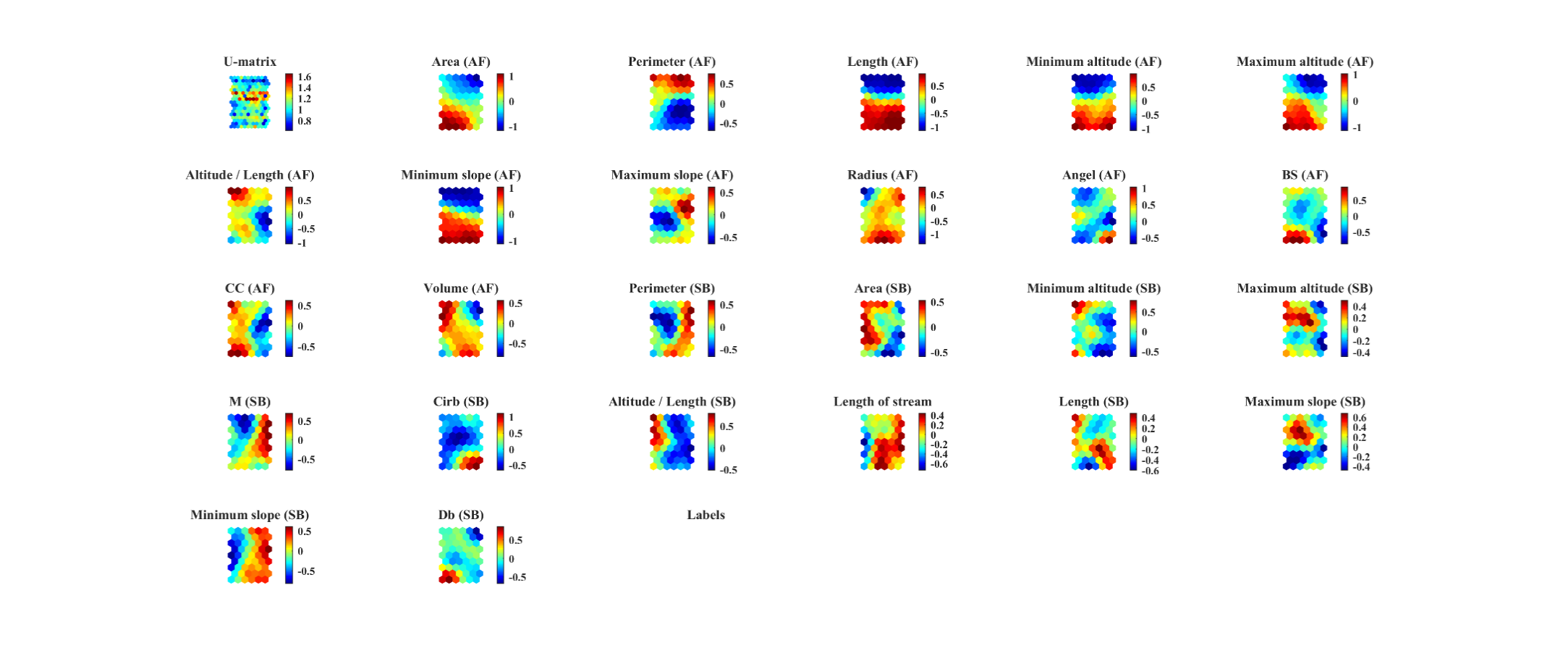

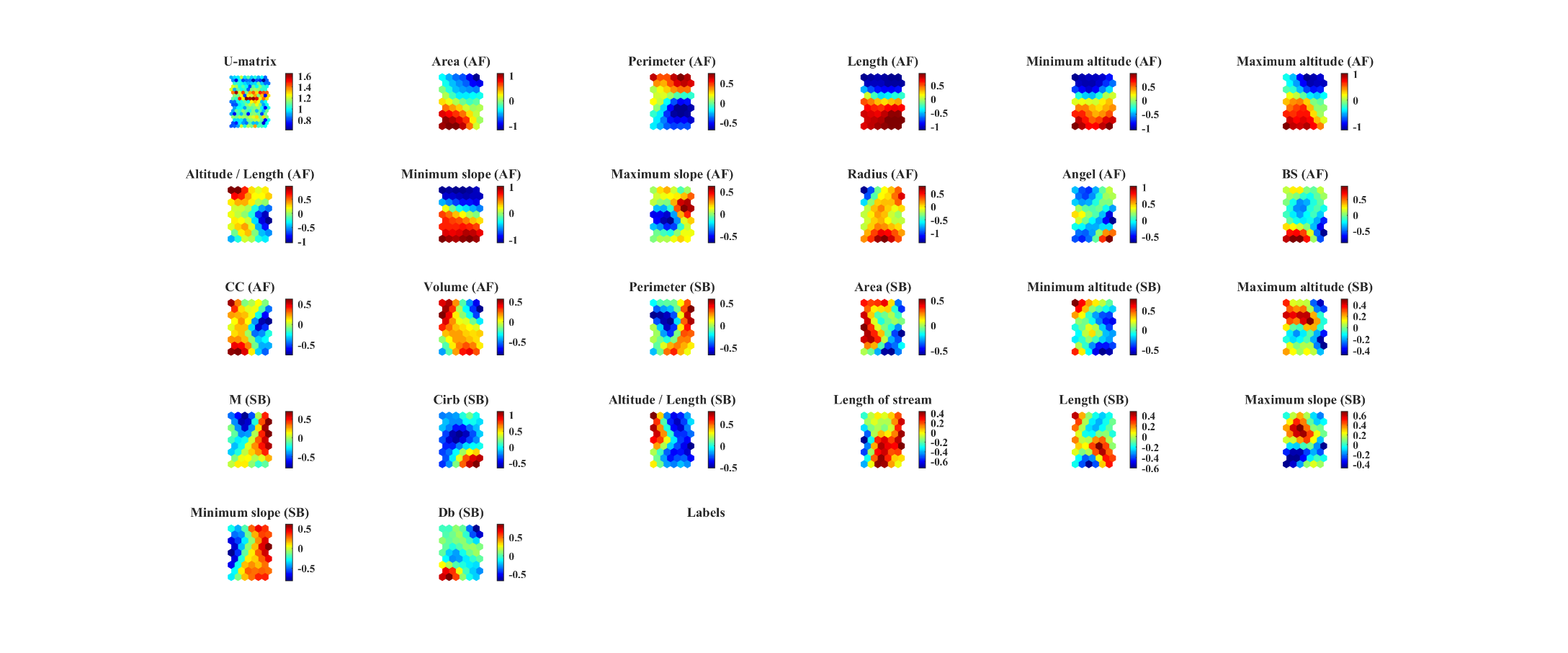

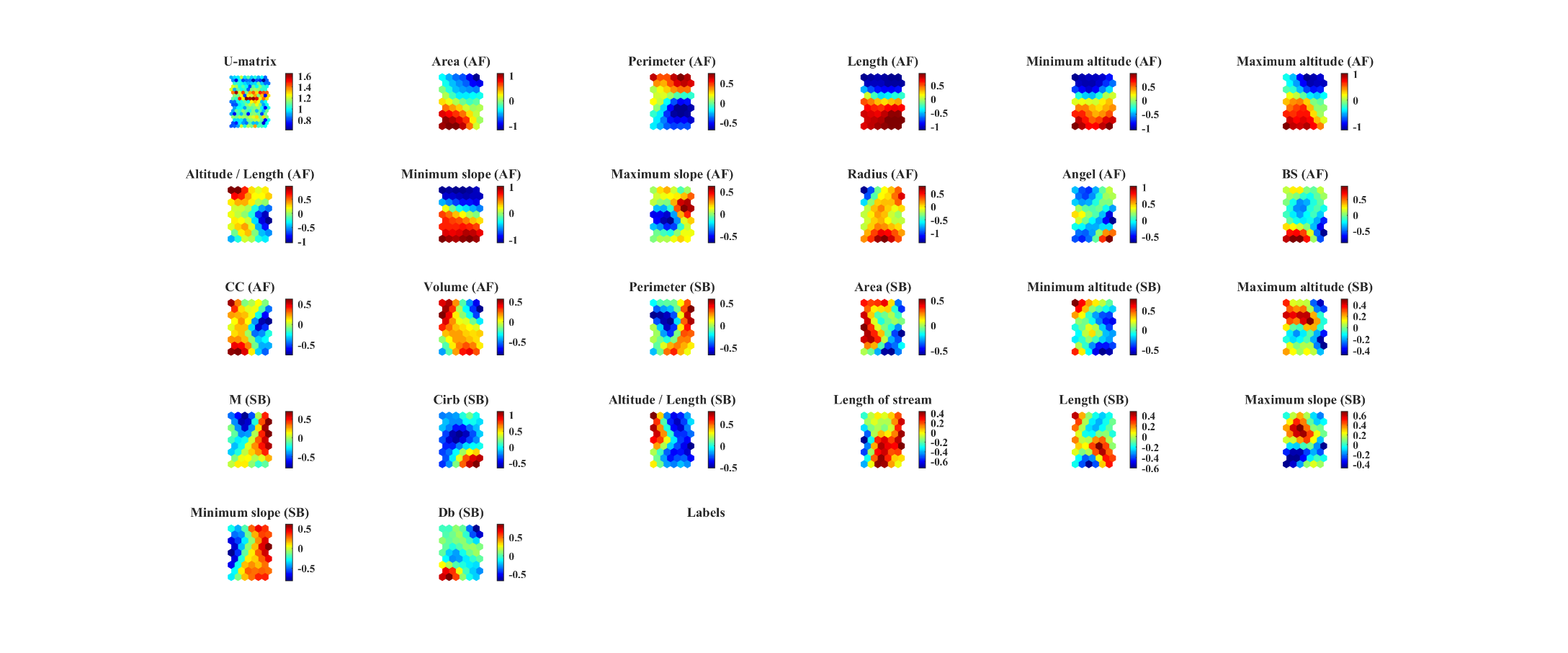

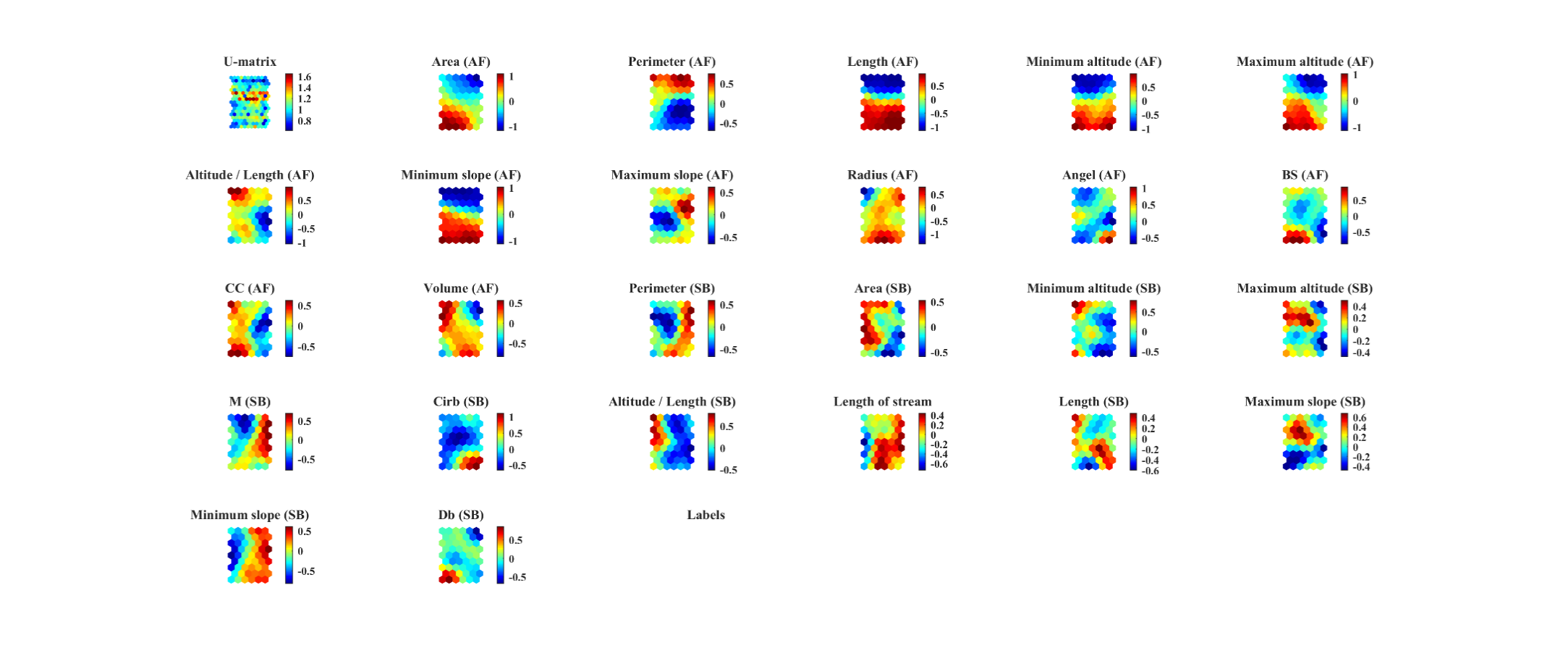

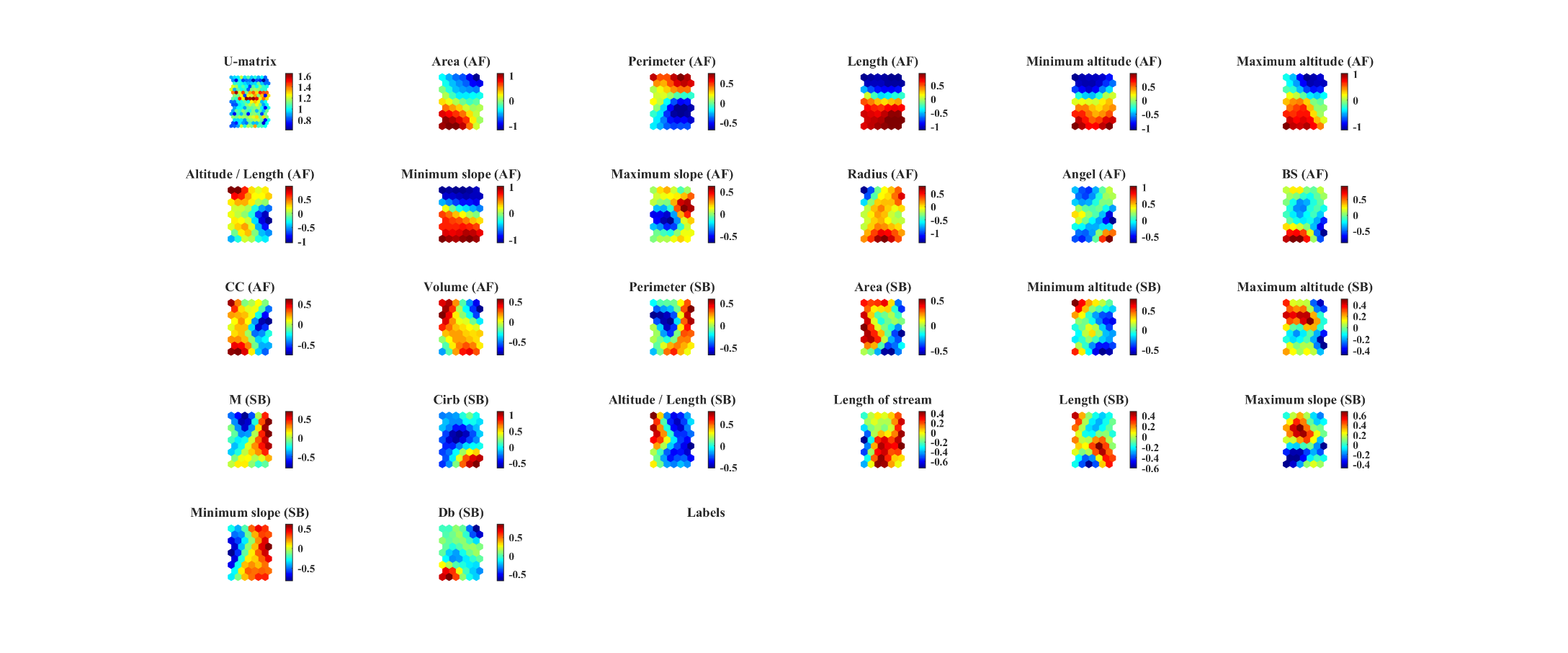


**(a)**


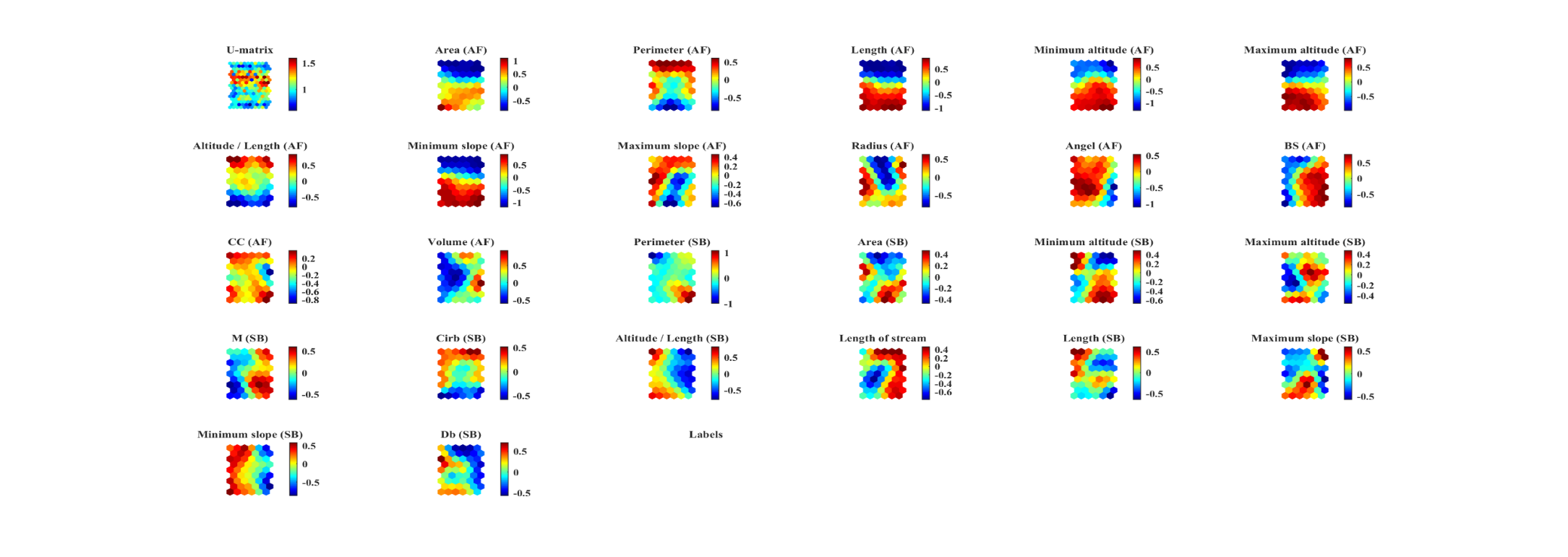

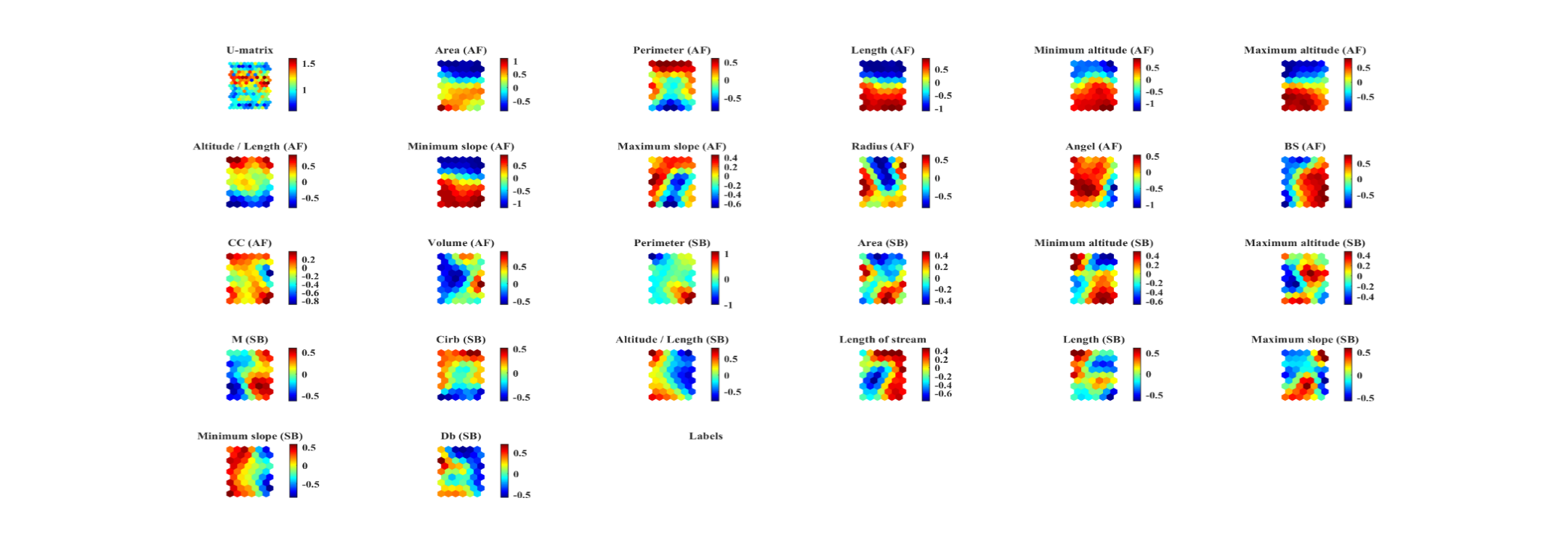

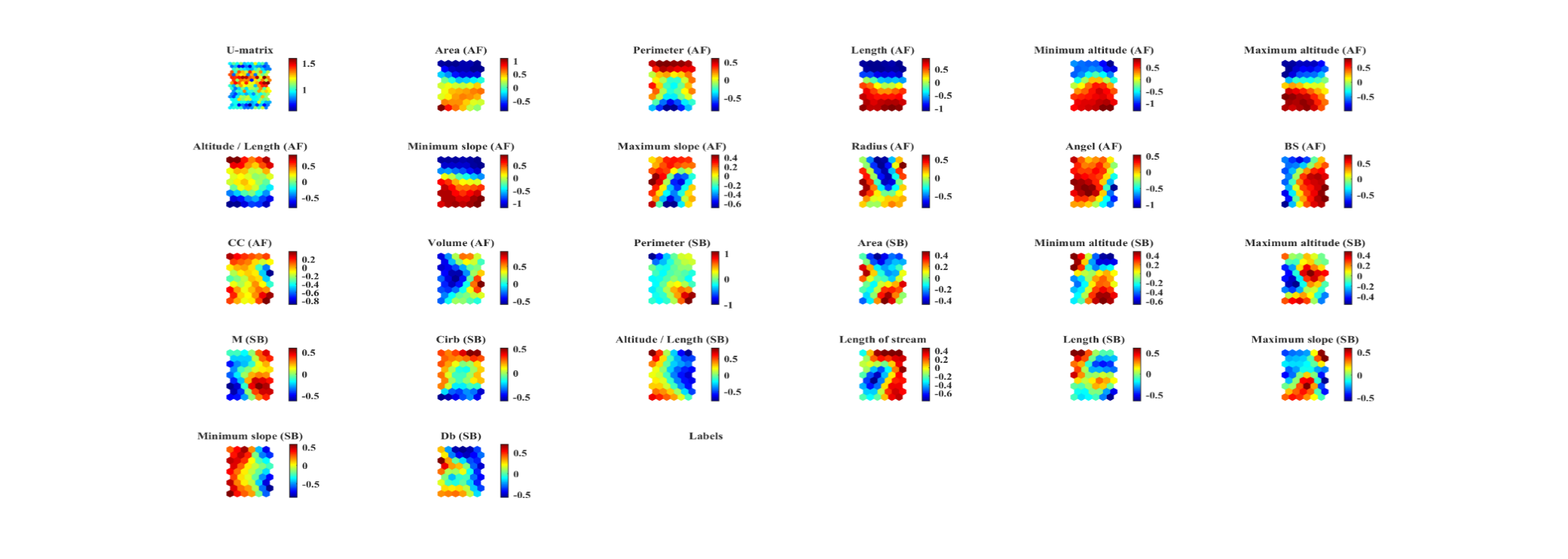

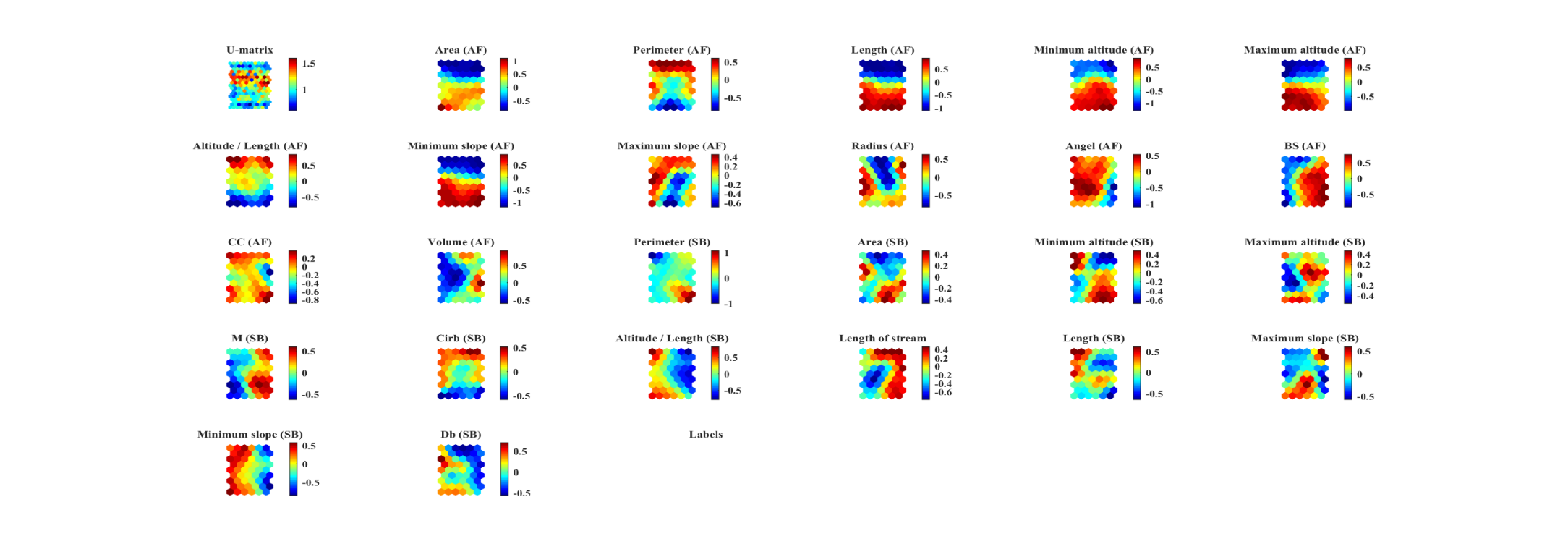

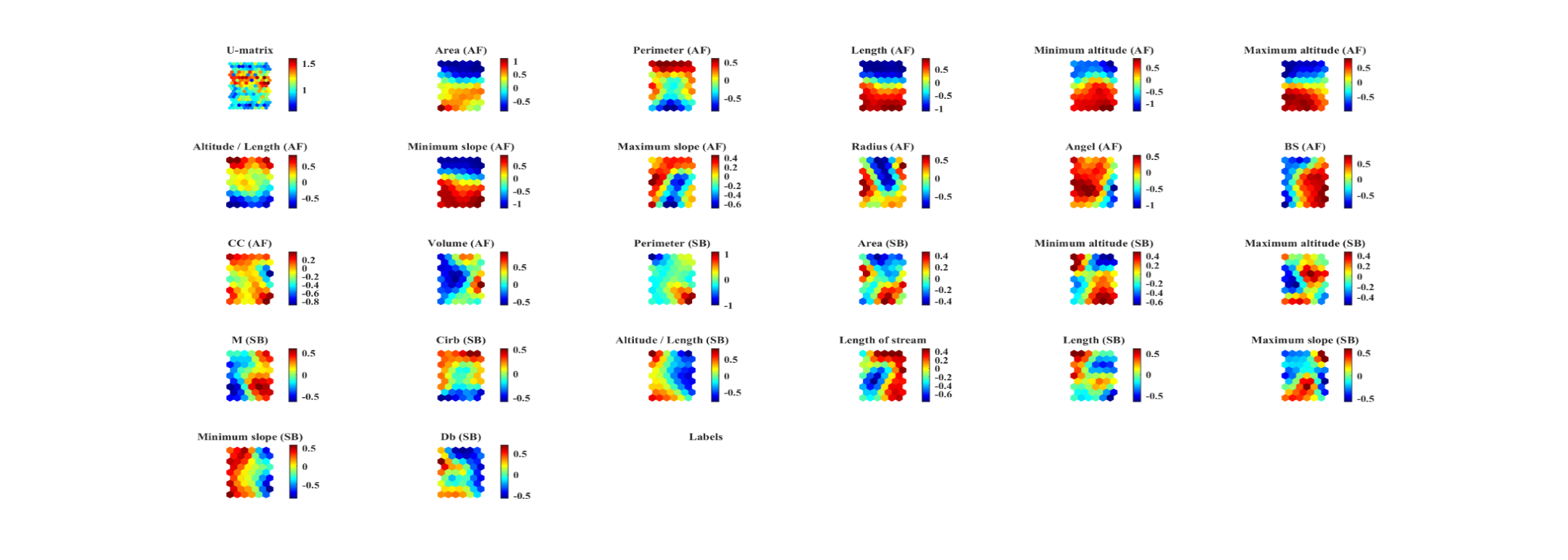

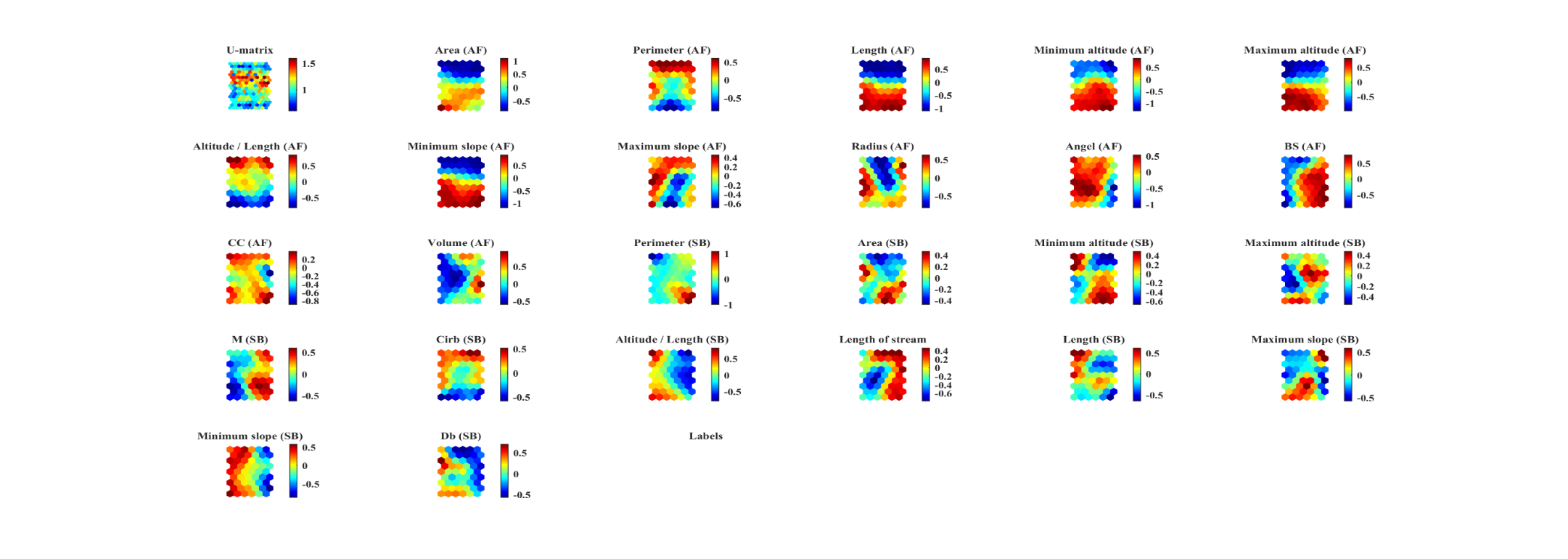


**(b)**

**Fig. S3.** SOM visualization through U-matrix (a): lithology, (b): erosion
